# Pleural mesothelioma risk by industry and occupation: results from the Multicentre Italian Study on the Etiology of Mesothelioma (MISEM)

**DOI:** 10.1186/s12940-022-00869-5

**Published:** 2022-06-18

**Authors:** Enrica Migliore, Dario Consonni, Susan Peters, Roel C. H. Vermeulen, Hans Kromhout, Antonio Baldassarre, Domenica Cavone, Elisabetta Chellini, Corrado Magnani, Carolina Mensi, Enzo Merler, Marina Musti, Alessandro Marinaccio, Dario Mirabelli

**Affiliations:** 1 Cancer Epidemiology Unit, CPO Piemonte and University of Turin, Turin, Italy; 2 Interdepartmental Centre G. Scansetti for Studies On Asbestos and Other Toxic Particulates, University of Turin, Turin, Italy; 3 Occupational Health Unit, Fondazione IRCCS Ca’ Granda Ospedale Maggiore Policlinico, Milan, Italy; 4 Department of Environmental Epidemiology, Institute for Risk Assessment Sciences, Utrecht University, Utrecht, The Netherlands; 5 Occupational Medicine Unit, Careggi University Hospital, Florence, Italy; 6 Interdisciplinary Department of Medicine, Section of Occupational Medicine “B. Ramazzini”, University of Bari, Bari, Italy; 7 Unit of Environmental and Occupational Epidemiology, Cancer Prevention and Research Institute, Florence, Italy; 8 Department of Translational Medicine, Unit of Medical Statistics and Cancer Epidemiology, University of Eastern Piedmont and CPO Piemonte, Novara, Italy; 9 Occupational Health Unit, Department of Prevention, Padua, Italy; 10 Unit of Occupational and Environmental Epidemiology - Italian Mesothelioma Register, Department of Occupational and Environmental Medicine, Epidemiology and Hygiene, Italian Workers’ Compensation Authority (INAIL), Rome, Italy

**Keywords:** Pleural mesothelioma, Asbestos, Occupational exposure, Exposure assessment, Case–control study

## Abstract

**Background:**

The Italian mesothelioma registry (ReNaM) estimates mesothelioma incidence and addresses its etiology by assessing cases’ exposures but cannot provide relative risk estimates.

**Objectives:**

i) To estimate pleural mesothelioma relative risk by industry and occupation and by ReNaM categories of asbestos exposure; and ii) to provide quantitative estimates of the exposure–response relationship.

**Methods:**

A population-based mesothelioma case–control study was conducted in 2012–2014 in five Italian regions. Cases and age and gender frequency-matched controls were interviewed using a standard ReNaM questionnaire. Experts coded work histories according to international standard classifications of industries/occupations and assigned asbestos exposure according to ReNaM categories. Job codes were further linked to SYN-JEM, a quantitative job-exposure matrix. Cumulative exposure (CE, f/mL-years) was computed by summing individual exposures over lifetime work history. Unconditional logistic regression analyses adjusted by gender, centre and age were fitted to calculate odds ratios (OR) and 95% confidence intervals (CI).

**Results:**

Among men we observed increased risks of mesothelioma in many industries and associated occupations, including: asbestos-cement (OR = 3.43), manufacture of railroad equipment (OR = 8.07), shipbuilding and repairing (OR = 2.34), iron and steel mills (OR = 2.15), and construction (OR = 1.94). ORs by ReNaM exposure categories were as follows: definite/probable occupational exposure (OR = 15.8, men; OR = 8.80, women), possible occupational (OR = 2.82, men; OR = 3.70, women), sharing home with an exposed worker (OR = 2.55, men; OR = 10.3, women), residential (OR = 2.14, men; OR = 3.24, women). Based on SYN-JEM, mesothelioma risk increased by almost 30% per f/mL-year (OR = 1.28, CI 1.16–1.42).

**Conclusions:**

Out study involved five regions with historically different types and levels of industrial development, encompassing one third of the Italian population and half of Italian mesothelioma cases. As expected, we found increased pleural mesothelioma risk in the asbestos industry and in trades with large consumption of asbestos materials. Clear associations were found using both qualitative (ReNaM classifications) and quantitative estimates (using SYN-JEM) of past asbestos exposure, with clear evidence of an exposure–response relationship.

**Supplementary Information:**

The online version contains supplementary material available at 10.1186/s12940-022-00869-5.

## Background

All uses of asbestos have been banned since 1999 in the European Union, and in some member states the ban was issued much earlier [1]. The incidence of malignant mesothelioma (MM) – the neoplasm most strongly associated with asbestos – showed a decline in Sweden, where the first ban had been issued in 1975 for crocidolite [2]. In the other European countries MM incidence was still increasing at the turn of century, although less steeply than in the past [1]; a recent decreasing trend in mortality has been observed in some Northern and Western European countries like Sweden and The Netherlands, but not yet in Italy [3]. Furthermore, it is widely acknowledged that asbestos remaining in place may result in exposure for workers engaged in maintenance and renovation of buildings and industrial plants, as well as for members of the general population due to the presence of weathering asbestos-containing materials. The epidemiological surveillance of MM remains, therefore, of interest.

In Italy use of asbestos was forbidden in 1992 and a National Mesothelioma Registry (ReNaM) was started in 1993. Registration is accomplished by its regional operating centres (centres, from now on). ReNaM centres identify incident MM cases in their regional populations, systematically collect information about their occupational and non-occupational exposures to asbestos, carry out the exposure assessment and feed data to ReNaM. Statistics are periodically published describing the proportion of cases with recognized occupational exposures and their distribution by industry and occupation [4]. Interestingly, it was found that about 10% of all Italian cases had no recognized exposure at work but had been exposed to asbestos in non-occupational settings [5].

The ability to describe etiology characterizes ReNaM and differentiates it from general cancer registries. However, exposure data being restricted to cases, ReNaM cannot provide risk estimates by industry and occupation. Furthermore, previously unrecognized opportunities and circumstances of exposure have been identified, but only when they gave rise to obvious clusters of cases in specific factories, industries or small areas [6–10]. To overcome such limitations a population-based case–control design would be instrumental, following the example of the French Programme National de Surveillance des Mésothéliomes (PNSM) [11]. To assess its feasibility and evaluate its potential results we conducted a population-based case–control study. The specific aims of this study were to i) estimate pleural mesothelioma relative risk by industry and occupation and by ReNaM categories of asbestos exposure; and ii) provide quantitative estimates of the exposure–response relationship.

## Methods

The study included pleural MM cases with histological confirmation of diagnosis. Cases were enrolled into the study as soon as they were identified. Given the constraints imposed by the funding body on the time-extension of the study (two years and six months, from October 2012 to March 2015), cases occurring during the recruitment period but registered and, thus, interviewed later were excluded. Only centers that could take advantage of a rapid alert system for the identification of incident cases were, therefore, involved. Five centers, Piedmont, Lombardy, Veneto, Tuscany and Apulia, participated in the study; in Piedmont the source population was limited to residents in the province of Turin and the local health district of Casale Monferrato, whereas in Veneto recruitment was restricted to residents in the provinces of Venice and Padua (Table 1).

**Table 1:** Characteristics of cases and controls, the MISEM study, 2012–2015, Italy

	Men				Women			
	Cases		Controls		Cases		Controls	
	N	(%)	N	(%)	N	(%)	N	(%)
Total	463	(48.6)	490	(51.4)	163	(41.7)	228	(58.3)
Age (years):								
< 50	9	(1.9)	24	(4.9)	4	(2.5)	18	(7.9)
50–54	17	(3.7)	30	(6.1)	6	(3.7)	7	(3.1)
55–59	30	(6.5)	45	(9.2)	10	(6.1)	17	(7.5)
60–64	42	(9.1)	54	(11.0)	12	(7.4)	24	(10.5)
65–69	90	(19.4)	117	(23.9)	16	(9.8)	43	(18.9)
70–74	111	(24.0)	102	(20.8)	46	(28.2)	51	(22.4)
75–79	92	(19.9)	62	(12.7)	30	(18.4)	25	(11.0)
80–84	57	(12.3)	38	(7.8)	29	(17.8)	25	(11.0)
85 +	15	(3.2)	18	(3.7)	10	(6.1)	18	(7.9)
Centre:								
Piedmont	167	(36.1)	159	(32.5)	67	(41.4)	108	(47.4)
Lombardy	146	(31.5)	141	(28.8)	59	(36.2)	62	(27.2)
Veneto	55	(11.9)	106	(21.6)	19	(11.7)	37	(16.2)
Tuscany	65	(14.0)	26	(5.3)	14	(8.6)	7	(3.1)
Apulia	30	(6.5)	58	(11.8)	4	(2.5)	14	(6.1)
Interview:								
Direct	353	(76.2)	454	(92.7)	97	(59.5)	217	(95.2)
Next of kin	110	(23.8)	36	(7.4)	66	(40.5)	11	(4.8)
Blue collar jobs:								
Ever	403	(87.0)	361	(74.0)	112	(68.7)	130	(57.0)
Never	60	(13.0)	127	(25.9)	50	(30.7)	98	(43.0)
Always	238	(51.4)	164	(33.5)	79	(48.5)	80	(35.1)
Number of jobs:								
Mean	3.86		3.47		2.48		2.42	
Std deviation	2.14		2.05		1.68		1.66	

Population controls were randomly selected from the regional rosters of citizens registered with the National Health Service. Such lists largely coincide with residents, as they are based on data from the municipal registrar offices. The average update lag is about six months, so control selection was carried out at mid-year of the recruitment year (in Piedmont and Veneto of the first recruitment year) of cases. Controls were frequency matched to the expected gender- and age-distribution of cases.

Personal interviews were carried out by trained interviewers who were blind to the case/control status of the study subjects. In Lombardy and Tuscany organizational and administrative constraints led, however, to conduct separate series of interviews for cases and controls: whereas those of cases were performed by occupational health officials of the Local Health Authorities, those of controls were carried out by ad hoc interviewers.

Occupational and non-occupational circumstances that could have entailed exposure to asbestos were investigated using the standardized ReNaM questionnaire, administered by trained interviewers to all study subjects. A next of kin was interviewed in case of participants’ death or when their conditions prevented direct interviews [5]. Lifetime occupational histories were collected for cases and controls, supplemented by job-specific modules allowing the description of the working environment, the tasks carried out by the interviewees or in their presence, the general ventilation and local exhaust systems. Full residential histories were also reconstructed, including residential addresses, the presence of industrial premises including iron and steel foundries, chemical plants, power plants, and asbestos-cement industries in proximity to residences were collected, as well as details on the characteristics of residential buildings, including the presence of prefabricated structures, asbestos-cement structures (walls and roofs), insulating materials. Lastly, the occupational histories of family members were collected.

Work histories were coded by Regional Operating Centre (COR) experts blind to the case–control status of study subjects. For the purposes of this study, industries and job titles were coded according to the International Standard Industrial Classification of Economic Activities (ISIC), second revision 1971 [12] and the International Standard Classification of Occupations (ISCO), second revision 1968 [13]. Job histories were truncated to the year of diagnosis (for cases) and enrolment (for controls). Industries were coded at the finest possible detail: the four- and five-digit level in ISIC and, respectively, ISCO classifications. Three-digit level ISCO codes to be used in data analysis were then generated by truncation of five-digit codes.

Quantitative indices of exposure to asbestos were obtained by merging the list of coded employment periods with the estimates of exposure provided by SYN-JEM [14]. SYN-JEM is a quantitative job-exposure matrix for five occupational respiratory carcinogens developed in the framework of the SYNERGY study, coordinated by the International Agency for Research on Cancer (IARC) [15]. In SYN-JEM exposure to asbestos has been estimated in fibre per millilitre units (f/mL) by country, region, historical period and job, where jobs have been classified according to ISCO 1968 [13]. Merging the job history with SYN-JEM provided yearly quantitative estimates of exposure, allowing the calculation of: (i) cumulative exposure (CE, in f/mL-y), (ii) duration of exposure, and (iii) average exposure intensity, by dividing CE by duration. Unlagged and lagged (10-, 20- and 30-year lag) indices were calculated.

Cases and controls also underwent the ReNaM standard assessment of the exposure source and probability [5]. Local experts, usually industrial hygienists or occupational health physicians with specific knowledge of the local past uses and natural occurrence of asbestos, performed such assessment.

Every job was assessed based on all the information available to the experts, such as interview data, previous interviews to possible earlier cases observed in the same workplaces, direct knowledge of industries and workplaces. Exposure probability was classified as definite (use of asbestos described at interview or already known to experts), probable (asbestos certainly used in the plant, but use by the interviewee unknown), possible (recognized asbestos use in the job or industrial activity, but unknown whether in the plant), unlikely (use of asbestos not described at interview and unknown to experts) or unknown (information inadequate to classify the job into any of the previous categories).

The possibility of para-occupational exposure (familial exposure in the ReNaM classification) was evaluated by examining the occupational histories of relatives (parents, siblings, spouses etc.) during the periods when they shared home with study subjects. Exposure was classified as “familial” (living with an occupationally exposed person), unlikely or unknown.

All residences, including those held habitually during holidays, were assessed for residential proximity to industrial or natural asbestos sources, based on their address and the spatial distribution of known sources. Exposure was classified as “residential” (residence in proximity – based on raters’ judgement – to one or more identified sources of asbestos pollution), unlikely or unknown.

Other non-occupational exposures may have occurred, such as the presence of asbestos-containing materials in the home environment or at school, the use of asbestos or the intervention on asbestos-materials during home maintenance and repairs or leisure-time activities, all of which were investigated in specific sections of the questionnaire. Exposure was assessed as “other non-occupational” (when any of such circumstances has occurred), unlikely or unknown.

According to the ReNaM guide-lines for exposure assessment [16], when multiple circumstances and routes of exposure are present, as is often the case, the individual overall classification is determined by the most severe exposure category ever experienced by a study subject, which is conventionally established according to the gradient (from most to less severe): occupational, familial, residential, other non-occupational exposures.

Odds ratios (OR) and 95% confidence intervals (CI) by industry and job – ever vs. never employment – were calculated by unconditional logistic regression adjusting by centre (as in Table 1) and 5-year age class (from < 50 to ≥ 85 years) for men and women separately. We used full, four-digit ISIC codes for industries and three-digit ISCO codes for occupations.

ORs and CIs were also calculated: (i) by gender and ReNaM exposure category (individual overall classification, as above), adjusted by centre, age-class (as above) and type of interview (direct or proxy), and (ii) by gender and CE, adjusted by centre, age-class, type of interview and binary indicator variables for ever-exposure in non-occupational settings (i.e.: familial, residential or other non-occupational circumstances).

In the analyses by ReNaM exposure category, we used as reference the combination of unlikely and unknown exposures. The unlikely exposure category could have been a better choice, but it could not be used because of the relatively small number of cases and control so classified. We also combined definite and probable occupational exposures, as the number of cases and controls classified as probably exposed was small, especially among women.

CE was modeled both as a categorical and a continuous variable. In the first case, CE categories were built based on the CE distribution among exposed controls, considering as cut-off values the median (exposed below and above the median) and tertiles (first, second and third CE tertile). In the latter case, either the untransformed or the natural-log transformed variables were used.

Unlagged and lagged (at 10-, 20- and 30-years lag) analyses were carried out. All analyses were performed also by combining men and women, adding gender to the model. Analyses by ReNaM exposure category were replicated also by using as reference only unlikely exposures, and by lumping together all occupational exposures or, on the opposite, by separating definite, probable and possible exposures. Models were compared by calculating the Akaike information criterion (AIC).

We additionally fit a cubic spline model (with the same adjustment variables) allowing the slope of the function to change at predefined bending points (five knots at 10/25/50/75/90 percentiles), to better capture and describe the features of the exposure–response association, using untransformed and natural-log transformed CE, for men and women separately and considering the above specified lags (0, 10, 20 and 30 years).

Sensitivity analyses were carried out by replicating all models based on the ReNaM exposure indices and CE after (a) leaving out centres one at a time, (b) additional adjustment for the condition of blue-collar (BC) worker, and (c) restriction to BC workers. To this purpose, the study subjects were classified as ever vs. never holding a BC job. All occupations in the ISCO classification associated with a code equal to 5.10.00 or higher were considered BC jobs and included working proprietors, farmers and manual workers in industry and services.

All analyses were carried out with Stata 16 (Stata Corp. 2019, College Station, TX, USA).

The study was approved by the Internal Review Board of the coordinating centre (Cancer Epidemiology, Turin). Centre participation was approved by their respective Internal Review Boards. Participants gave their written, informed consent before interview.

## Results

In Table 1 the main characteristics of cases and controls are described. Supplementary Table S1 shows the distribution of cases and controls by centre, along with the size of target populations and recruitment periods. Direct interviews were obtained for 450 of the 626 cases eligible for the study (71.8%), and for 671 of the 718 controls (93.5%). For the remaining cases and controls, information was obtained from relatives (mainly from spouses, sons or daughters). Seventy-four percent of cases were men, with a mean age of 70.6 years (sd = 9.0). Cases and controls were very similar regarding the number of reported jobs (3.86 vs. 3.47, respectively, in men and 2.48 vs. 2.42 in women).

**Fig. 1:**
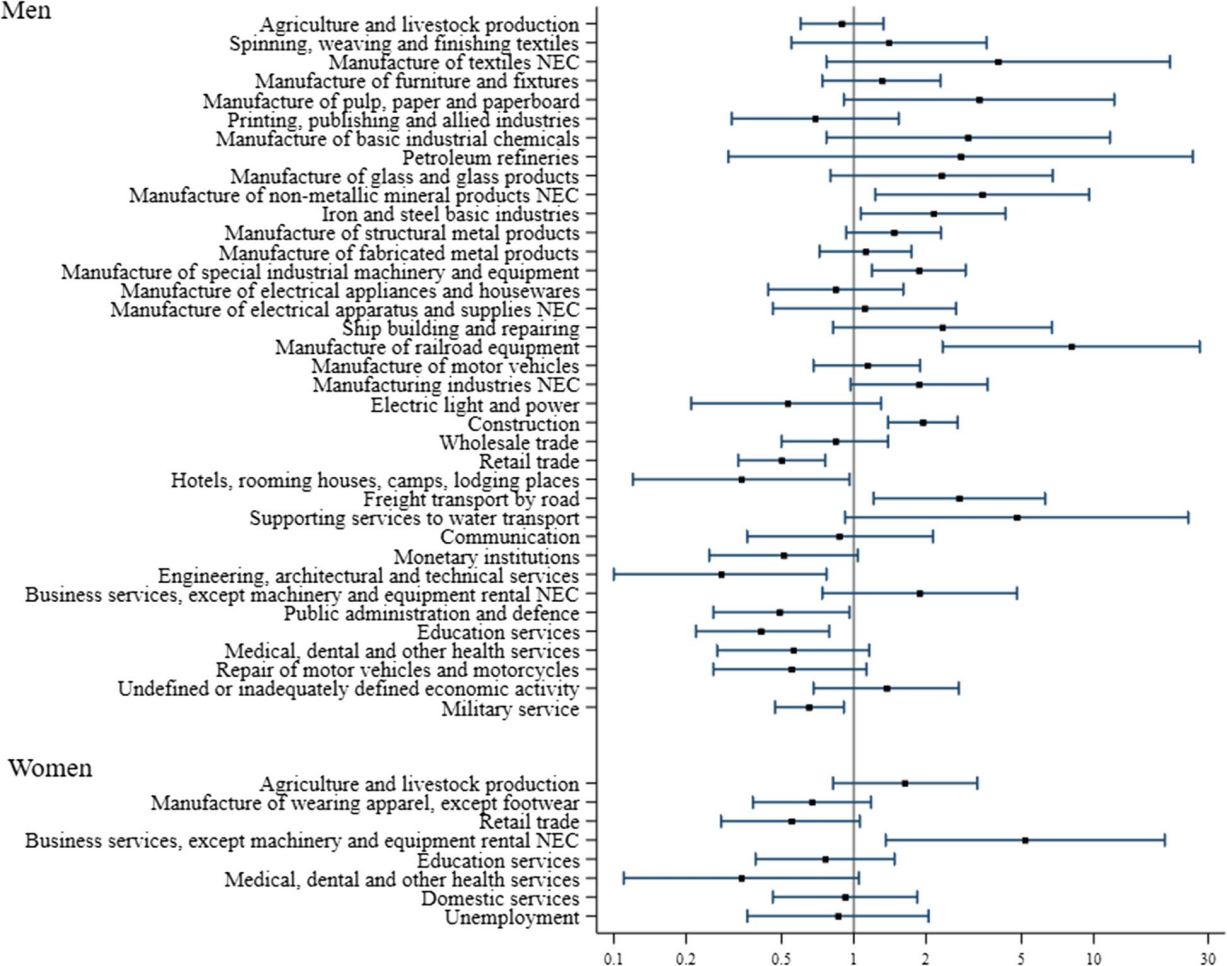
Odds ratios and 95% confidence intervals for pleural mesothelioma by gender and industry, according to the International Standard Industry Classification (ISIC, 4-digit codes), 1971 – Industries with at least 20 exposed cases and controls—the MISEM study, 2012–2015, Italy

**Fig. 2:**
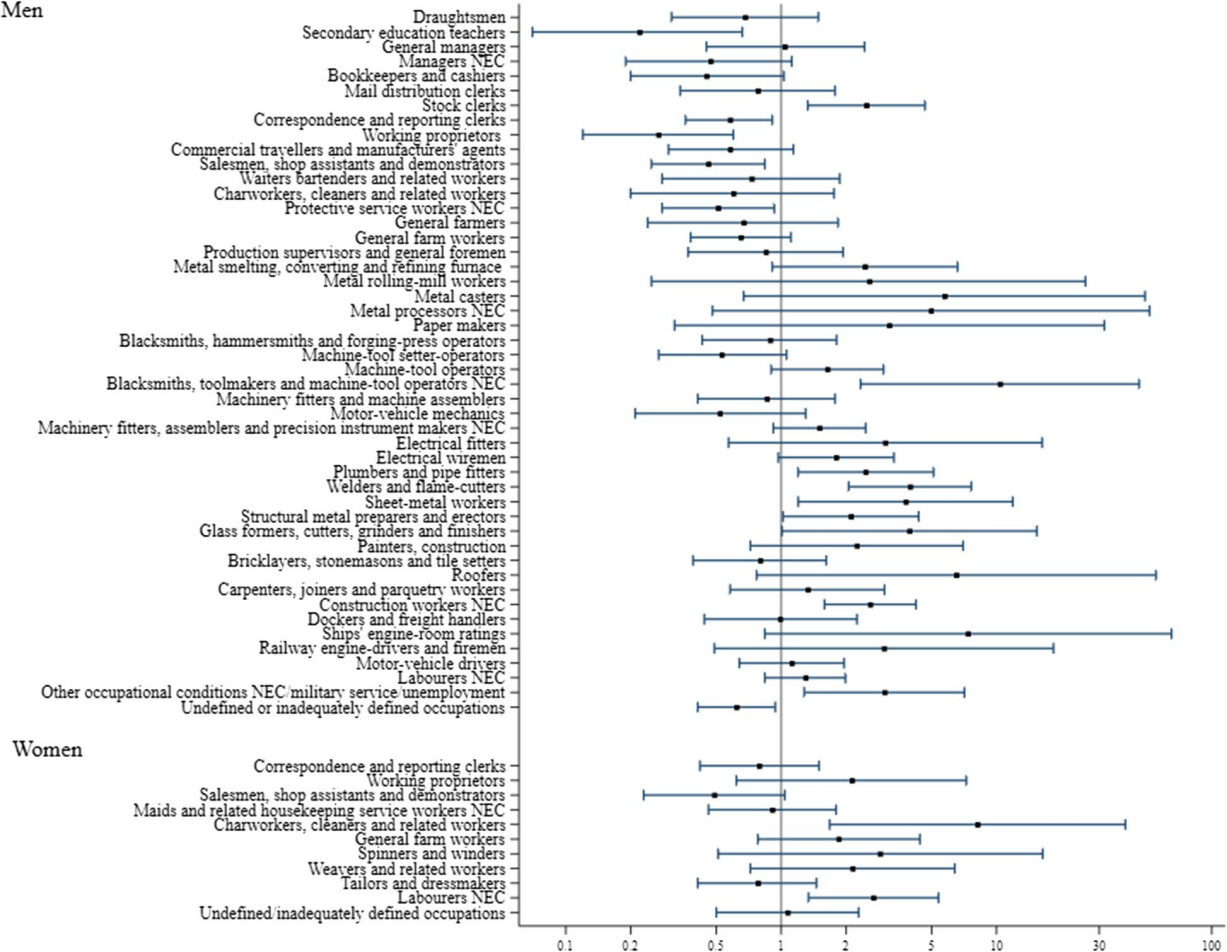
Odds ratios and 95% confidence intervals for pleural mesothelioma by gender and occupation, according to the International Standard Code of Occupations (ISCO, 3-digit codes), 1968 – Occupations with at least 20 exposed cases and controls—the MISEM study, 2012–2015, Italy

Selected results for men and women by industry (ordered by ISIC 4-digit codes) and job (ordered by ISCO 3-digit codes) are plotted in Figs. 1 and 2. The full set of results for industries and occupations with at least three exposed cases is provided in Supplementary Tables S2 and S3.

No study subject reported employment in asbestos mining or in associated occupations. Asbestos transformation activities are lumped together in the ISIC classification under the rubric “Manufacture of non-metallic mineral products not elsewhere classified”, ISIC code 3699, for which we found a more than three-fold increase in pleural MM risk, among men; in women there were six exposed cases and no exposed controls.

Employment in various industries known to have entailed extensive use of insulation materials was associated with high ORs, namely: manufacture of railroad equipment (ISIC code 3842), ship building and repairing (ISIC code 3841), chemical industry (in particular: manufacture of basic industrial chemicals, ISIC code 3511), oil refineries (ISIC code 3530), iron and steel mills (ISIC code 3710), glass industry (ISIC code 3620). We found excess risk also in jobs specifically associated with some of these industries, such as blacksmiths (ISCO code 839), metal furnacemen (ISCO code 721), rolling mill workers (ISCO code 722), metal casters (ISCO code 724), metal processors not elsewhere classified (ISCO code 729), as well as glass formers and cutters (ISCO code 891).

Construction industry was associated with an approximately two-fold MM risk in men and in women (albeit with a rather large CI). Among men this was by far the largest exposure group with 119 exposed cases. In addition, many occupations in the construction industry were also associated with increased ORs: electrical fitters (ISCO code 851), plumbers and pipe-fitters (ISCO code 871), roofers (ISCO code 953), general construction workers (ISCO 959) and construction painters (ISCO code 931). The manufacture of special industrial machinery and equipment was also associated with an almost two-fold increase in MM risk among men (ISIC code 3824), and even higher risks were entailed by related jobs like welders and flame cutters (ISCO code 872), sheet metal workers (ISCO code 873) and structural metal preparers and erectors (ISCO code 874) as well as plumbers and pipe-fitters (ISCO code 871), who may be employed also in this trade.

Interestingly, MM risk in men was high after employment in freight transport by road (ISIC code 7114) and in water transport (OR = 3.31; CI 1.12–9.84, not shown in Fig. 1 and in Supplementary table S2 as this trade corresponds to a three-digit ISIC code: 712), including supporting services to water transport (ISIC code 7123). Correspondingly, ship’s engine room personnel (ISCO code 982) and railway engine drivers (ISCO code 983) had increased ORs.

MM risk was elevated among men also in manufacture of textiles not elsewhere classified (ISIC code 3219), which mainly included production of felts and mattresses, in the pulp and paper industry (ISIC code 3411) and in the poorly defined group of manufacturing industries not elsewhere classified (ISIC code 3909). As to occupations, paper makers in men (ISCO code 734) and spinners/winders and weavers in men and women (ISCO codes 752, 754) had increased ORs – with wide CIs.

Stock clerks (ISCO code 391) and occupational conditions not corresponding to any ISCO item – such as military service and unemployment – were at high risk among men, in addition to the jobs previously mentioned in relation with their corresponding industries. Thirty-five male cases and 16 controls reported, respectively, 39 and 21 employment periods as stock clerks, out of which 30 and 12 were spent in industrial settings where the use of asbestos-containing materials was possible or even likely, such as the chemical, rubber and plastics industry, steel mills and the construction of industrial machinery and equipment.

Among women, the only trade associated with a clear-cut increase in MM OR was that of business services not elsewhere classified (ISIC code 8329). This is a large group, which may include businesses as different as cleaning services and marketing. The ten women cases in our study reported 20 employment periods in ISIC 8329, 19 of which were spent in cleaning services, while the three controls had 3 employment periods, one of which in cleaning services. It is worth mentioning that such cleaning services had been often described at interview as industrial cleaning services, and in at least two cases in work-settings well known for having entailed exposure to asbestos. Consistently with these results, charworkers, cleaners and related workers (ISCO code 552) was the female job code with the largest MM risk.

ReNaM exposure indices for occupational and non-occupational exposures were strongly associated with increased risk of pleural MM in both genders (Table 2). Overall, for definite/probable occupational exposure, we found an OR of about 15 while possible occupational exposures also corresponded to a three-fold elevated risk. Elevated ORs were also found for familial exposures, especially in women. The results of the analyses in which occupational exposures had been either grouped (definite, probable and possible) or considered as distinct categories are reported, respectively, in Supplementary Tables S4 and S5. Comparing the AIC values for the corresponding, gender-specific models from Table 2 and Supplementary Tables S4 and S5 suggests that those from Table 2 fitted data better. Furthermore, in Supplementary Table S6 we report our findings from analyses using only unlikely exposures as the reference category, rather than combining them with unknown exposures: all ORs for the occupational and non-occupational exposure categories corresponding to those from Table 2 were slightly higher, and indeed the unknown exposure category itself was associated with an increased OR among men. All results in Table 2 and Supplementary Tables S4-S6 were obtained after allowing for a 10-years lag in the analyses. Unlagged and 20-years lagged results as well as the AIC values for corresponding models were very similar, whereas introducing a 30-year lag slightly reduced the OR point estimates, increasing the AIC values (results not shown).

**Table 2:** Number of cases and controls, odds ratio (OR) and 95% confidence intervals (CI) by modality of exposure, according to the Italian National Mesothelioma Register (ReNaM) classification. Reference category: unlikely/unknown exposure, lag 10 years – the MISEM study, 2012–2015, Italy

ReNaM exposure	Men and women				Men				Women			
	**Cases**	**Ctrls**	**OR**^a^	**CI**	**Cases**	**Ctrls**	**OR**^b^	**CI**	**Cases**	**Ctrls**	**OR**^b^	**CI**
Unlikely/unknown exposure	122	382	1.00	(ref)	67	248	1.00	(ref)	55	134	1.00	(ref)
Occupational, definite or probable	343	110	14.8	(10.3–21.2)	306	96	15.8	(10.5–23.8)	37	14	8.80	(3.66–21.2)
Occupational, possible	61	80	3.02	(1.93–4.75)	48	67	2.82	(1.68–4.72)	13	13	3.70	(1.34–10.3)
Familial	40	45	4.63	(2.67–8.02)	14	28	2.55	(1.15–5.62)	26	17	10.3	(4.10–26.1)
Residential	44	75	2.39	(1.42–4.03)	20	42	2.14	(1.07–4.28)	24	33	3.24	(1.33–7.86)
Other non-occupational	13	23	2.58	(1.17–5.72)	7	8	4.67	(1.49–14.6)	6	15	2.16	(0.64–7.29)
*P-Wald test gender interaction*			0.07									
AIC			1409.71				1002.86				416.62	

*AIC*:Akaike information criterion

^a^OR adjusted by centre, gender, age, and type of interview

^b^OR adjusted by centre, age, and type of interview

**Fig. 3:**
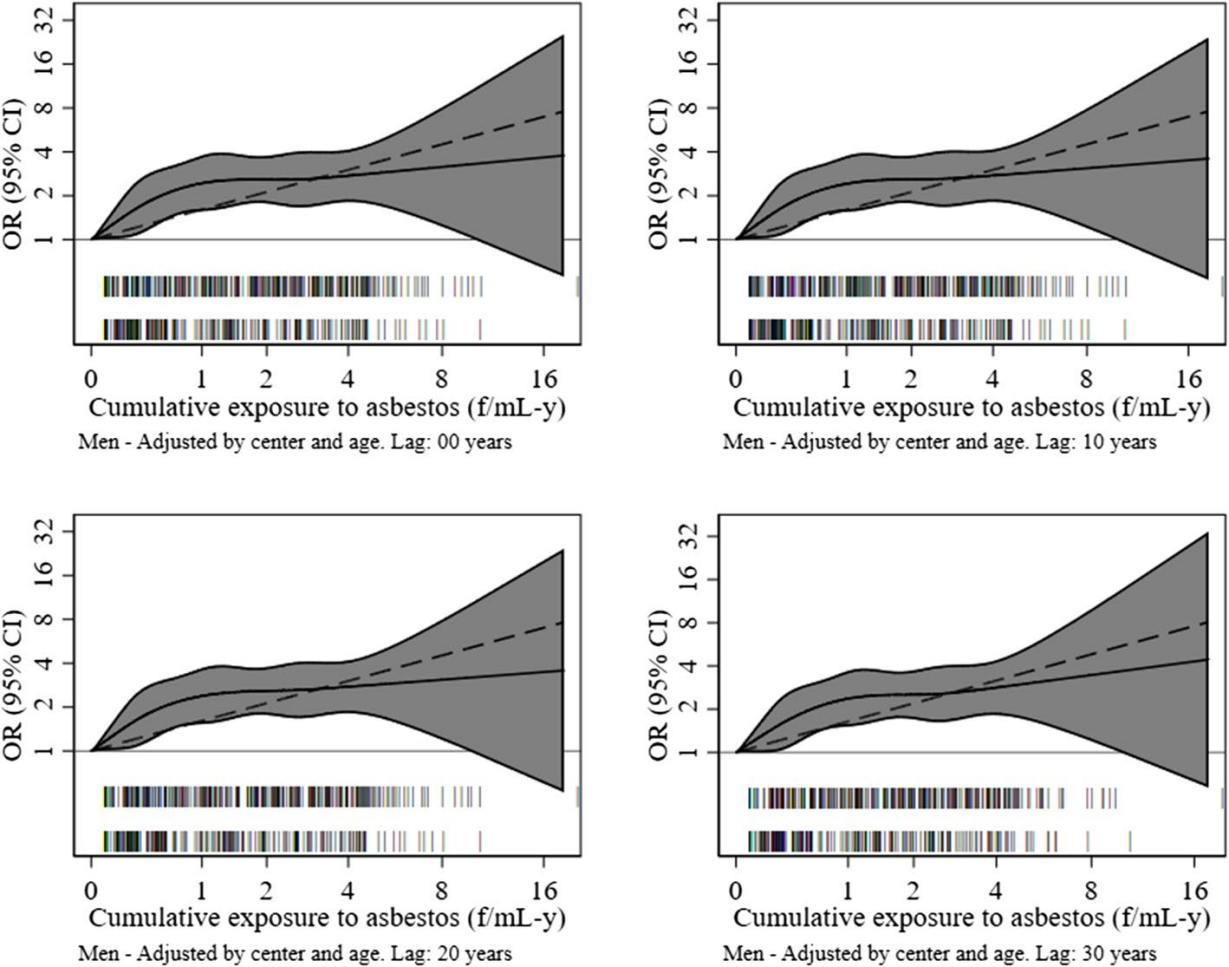
Odds ratios and 95% confidence intervals for pleural mesothelioma by cumulative exposure to asbestos (natural log-scale) modelled as a restricted cubic spline with five knots at 10/25/50/75/90 percentiles, men, lag10, 20 and 30 years – the MISEM study, 2012–2015, Italy

In Table 3 we describe the exposure–response relationship between MM risk and quantitative estimates of cumulative (occupational) exposure to asbestos. Exposed subjects had an approximately double risk of developing pleural MM. A positive linear trend (*P*-value < 0.001) for increasing OR was found in categorical analyses by CE. Spline modelling confirmed the association between CE and pleural MM, with the OR increasing steeply up to CE values around 1 f/mL-y and more slowly thereafter (Fig. 3).

**Table 3:** Number of cases and controls, odds ratio (OR) and 95% confidence interval (CI) by exposure (ever vs never) and cumulative exposure from SYN-JEM, unlagged and at lag 10, 20 and 30 years, men and women – the MISEM study, 2012–2015, Italy

			**Lag 0**		**Lag 10**		**Lag 20**		**Lag 30**	
**SYN-JEM exposure**	**Cases**	**Ctrls**	**OR**^a^	**CI**	**OR**^a^	**CI**	**OR**^a^	**CI**	**OR**^a^	**CI**
**Ever/never**										
Unexposed	278	446	1.00	(ref)	1.00	(ref)	1.00	(ref)	1.00	(ref)
Exposed	333	262	2.22	(1.70–2.91)	2.21	(1.69–2.90)	2.29	(1.74–3.00)	2.15	(1.64–2.82)
* P-Wald test gender interaction*			*0.27*		*0.28*		*0.28*		*0.59*	
AIC			1597.49		1595.26		1591.05		1609.33	
**Below/above median cumulative exposure (in f/mL-y)**										
Unexposed	278	446	1.00	(ref)	1.00	(ref)	1.00	(ref)	1.00	(ref)
< 0.86	120	131	1.71	(1.21–2.42)	1.71	(1.21–2.42)	1.84	(1.30–2.59)	1.74	(1.24–2.46)
≥ 0.86	213	131	2.66	(1.95–3.64)	2.65	(1.94–3.61)	2.68	(1.96–3.66)	2.52	(1.84–3.45)
* P-trend*			< *0.001*		< *0.001*		< *0.001*		< *0.001*	
* P-Wald test gender interaction*			*0.46*		*0.48*		*0.47*		*0.76*	
AIC			1593.89		1591.87		1589.08		1607.56	
**Tertiles of cumulative exposure (in f/mL-y)**										
Unexposed	278	446	1.00	(ref)	1.00	(ref)	1.00	(ref)	1.00	(ref)
< 0.34	64	87	1.35	(0.88–2.05)	1.34	(0.88–2.04)	1.45	(0.96–2.21)	1.21	(0.79–1.85)
0.34–1.62	118	87	2.52	(1.75–3.62)	2.53	(1.76–3.64)	2.59	(1.80–3.73)	2.48	(1.72–3.57)
> 1.62	151	88	2.65	(1.85–3.77)	2.62	(1.84–3.72)	2.69	(1.88–3.83)	2.64	(1.85–3.77)
* P-trend*			< *0.001*		< *0.001*		< *0.001*		< *0.001*	
* P-Wald test gender interaction*			*0.39*		*0.36*		*0.30*		*0.58*	
AIC			1591.96		1589.78		1587.16		1601.36	
**Cumulative exposure, continuos**										
Unexposed	278	446	1.00	(ref)	1.00	(ref)	1.00	(ref)	1.00	(ref)
Unit exposure: 1 f/mL-y	333	262	1.28	(1.16–1.42)	1.28	(1.16–1.41)	1.28	(1.16–1.42)	1.31	(1.18–1.46)
AIC			1603.43		1601.83		1600.27		1612.64	
**Log-cumulative exposure, continuos**										
Unexposed	278	446	1.00	(ref)	1.00	(ref)	1.00	(ref)	1.00	(ref)
Unit exposure: 1 log(f/mL-y + 1)	333	262	2.06	(1.62–2.61)	2.05	(1.61–2.60)	2.06	(1.62–2.61)	2.12	(1.65–2.72)
AIC			1594.23		1592.57		1590.98		1603.91	

*AIC* Akaike information criterion

^a^OR adjusted by centre, gender, age and type of interview

Results remained substantially unchanged in lagged analyses; they are shown in Table 3 alongside un-lagged findings: at 20-years lag the AIC was minimized, while widening the lag to 30 years increased it. At lag 20 the OR increased from 1.45 (CI 0.96–2.21) at < 0.34 f/mL-y to 2.59 (CI 1.80–3.73) at 0.34–1.62 f/mL-y and 2.69 (CI 1.88–3.83) at > 1,62 f/mL-y, while the OR per 1 f/mL-y of CE was 1.28 (CI 95% 1.16–1.42). Figure 3 shows that applying different lags did not modify the shape of the exposure–response relationship.

Gender-specific analyses were also carried out and results for men are shown in Supplementary Table S7: they were very similar to those in Table 3, with more evident trends in categorical analyses and almost identical results for those treating CE as a continuous variable.

Results from sensitivity analyses, replicating the same models as in Tables 2 and 3 are shown in Supplementary Tables S8 and S9 (by ReNaM exposure category and, respectively, by CE after additional adjustment for blue-collar status), and S10 and S11 (as before, restricted to blue-collar workers). Additional adjustment for blue-collar status brought little if any changes in results and model fit for the ReNaM exposure categories; ORs by CE category and unit exposure were slightly reduced. After restriction to ever blue-collar workers, the ORs associated with ReNaM exposure categories slightly decreased in men (but not in women) and confidence intervals were widened. ORs by CE category and by f/mL-year were reduced, but there remained a positive trend with increasing CE category and the risk did increase by f/mL-year.

## Discussion

In this population-based study, using different approaches, we found clear associations between MM risk and i) selected industries and occupations; ii) occupational and non-occupational exposures, classified according to ReNaM; and iii) occupational exposure indices calculated with a quantitative job-exposure matrix, SYN-JEM.

MISEM allowed for the first time the calculation of relative risk estimates by industry and occupation, providing evidence of increased risk for various trades which entailed widespread use of asbestos-containing materials and had relatively large prevalence in the general population. It also put to test the standard ReNaM exposure assessment, by computing risk estimates by ReNaM exposure index, showing that all ReNaM exposure categories were associated with substantially increased MM risk and that their conventional ranking broadly parallels MM risk. Lastly, linkage with SYN-JEM provided quantitative estimates of occupational exposures and exposure–response analyses showed a sub-linear relationship between CE and MM risk.

The Italian asbestos industry included all main types of asbestos products manufacturers. The first asbestos textiles factory opened around 1870 in the outskirts of Turin and several plants, mainly in Piedmont and Lombardy, were active up to the late 1980s. The oldest asbestos-cement plant was started in Casale Monferrato in 1907; asbestos-cement production peaked during the early 1970s, sustained by about 40 factories scattered all over Italy, a few of which continued activity until the asbestos ban in 1992. These industries, as well as the production of asbestos insulation boards, asbestos cardboard and asbestos brake and clutch linings, were included for the analysis in the “Manufacture of non-metallic mineral products not elsewhere classified” ISIC group (code 3699), which was associated with a clear-cut increase in MM risk—notwithstanding a most likely underestimation of the true risk for asbestos workers, due to the lack of specificity of this ISIC group, which comprises also non-asbestos industries. A further limitation is that, in 2012–2014, when MISEM cases and controls were recruited, a large number of former Italian asbestos workers had already died: asbestos-cement production was the single most important branch, estimated to use some 85% of all raw asbestos [17], and a pooled study of Italian cohorts with 13,076 workers from 21 asbestos-cement plants had registered 6626 decedents (52.7%) by the end of follow-up in 2012 [18]. The progressive shrinking of the pool of former asbestos workers made their exposure in the general population even rarer than originally; the limited number of exposed cases and controls in the study dataset accounts for the wide confidence interval of the risk estimate for men, and the absence of exposure controls among women.

Asbestos-containing materials and products (ACMs) found large industrial use and we observed increased MM risk in the trades where such ACMs had been mostly employed and in the associated occupations.

Manufacture of railroad equipment, ship building and repairing, chemical industry, oil refineries, iron and steel mills and the glass production industry are well known for the extensive use of insulation materials such as insulation blocks, asbestos felts and mattresses, and sprayed asbestos, whose fragility may entail substantial exposures during application and maintenance; indeed, when incompletely confined or during maintenance also production workers may get indirectly exposed. Such industries as well as various related jobs were associated with high ORs, and the same exposure patterns may be the explanation for similarly increased ORs in the manufacture of special industrial machinery and equipment and among welders and flame cutters, sheet metal workers and structural metal preparers and erectors.

We found elevated MM risk in the construction industry and in several occupations within it. This is in agreement with previous studies [19–24]. Notably, in this sector exposure continued (and is still continuing) after the asbestos ban due to presence of large quantities of still unremoved ACMs. Indeed, the majority of occupational exposures among male MM cases in Italy (15.5%) were estimated to have occurred in the construction industry [4].

Freight transport by road and water are two further economic activities for which exposure data from ReNaM had given a warning, as they accounted for about 6% of all asbestos occupational exposures [4]. Moreover, engine-room personnel were the most frequently exposure associated occupation. Our findings confirmed an increased MM risk.

Most occupational exposures for MM cases in women in Italy have been recorded in the textile industry and were assessed as due to the use of brake and clutch linings and structural fire-proofing and insulating materials [25].

Among men, stock clerks were at high MM risk. This apparently surprising result may be explained by their predominant employment in industrial settings where the use of asbestos-containing materials was likely (see results). Also, in women, the increased OR for charworkers, cleaners and related workers may be due to their engagement mainly in industrial cleaning services.

Risk estimates by ReNaM exposure category showed that ReNaM exposure indices for both occupational and non-occupational exposures were strongly associated with MM risk in men as well as in women. Occupational exposures (definite or probable) entailed the highest MM risk in men – as expected, as exposures at work are known to reach on average higher levels. However, this did not seem to be the case for women: even if their risk estimates have wide and overlapping confidence intervals, familial exposures were associated with the most elevated OR. This finding is consistent with other studies, such as the observed mortality from pleural malignancies (as a proxy for MM) among the wives of Casale Monferrato asbestos-cement workers: whereas these women had never been employed at the local asbestos-cement factory, they experienced an about 18-fold increase in mortality compared with the general female population. Such increase had been considered to be due to exposures at home, while accomplishing tasks such as cleaning workers’ clothes [26].

The results by ReNaM categories were confirmed when the analyses were repeated by changing the reference category: rather than combining unlikely and unknown exposures, only unlikely exposures were then used as reference. Point estimates were slightly increased, and the unknown exposure category itself was associated with a two-fold risk increase in men; this result suggests the unknown exposure category had been enriched by exposed individuals, which could be explained if experts engaged in exposure assessment had been conservative in their judgment, assigning exposures to this category when in doubt.

An additional result from MISEM is the assessment of the exposure–response relationship between asbestos and MM, which was made possible by associating the occupational histories of cases and controls with SYN-JEM, the job-exposure matrix developed for SYNERGY, a pooled analysis of case–control studies on lung cancer and occupation coordinated by IARC [14]. SYN-JEM had several features we deemed desirable. It estimated asbestos exposure by occupation according to the ISCO classification, which we also used in MISEM. Its estimates were data-driven, and Italian data were included in the ExpoSYN dataset from which SYN-JEM was modeled [27]. Furthermore, such estimates were: (i) region- even if not country-specific, so that we applied to MISEM the SYN-JEM estimates for Southern-Western European countries; and (ii) calendar-period-specific, accounting, thus, for exposure changes over the considerable time spanned by work histories.

We used CE as the simplest summary metric of life-long occupational exposures and assessed its association with MM risk using various modelling approaches: CE was positively associated with risk in categorical analyses as well as in those treating exposure as a continuous variable. Spline models showed that the OR increased steeply up to about 1 f/mL-y and more slowly at higher CE values. Non-linearity of the exposure–response relationship for pleural MM has been already suggested [28]. Our findings, however, seem to point to a stronger deviation from linearity than previously suggested. A contributory factor in our study may have been represented by type of distortion in the true shape of a linear dose–response described, among the others, by Steenland and coworkers [29]: they showed that, when CE is the metric of interest, random errors associated with assigning individual exposure levels based on group measurements or estimates – which of course happens when JEMs are applied – the shape is biased upwards in the middle range of CE and downwards at the highest CE values.

A further limitation of our analysis of the exposure–response relationship is that we could obtain quantitative estimates only for occupational exposures. We did, however, adjust our models for non-occupational exposures.

Finally, we want to mention several limitations associated with interview and response differences between cases and controls. The first one is that interviewers could not be blinded to the case/control status in Lombardy and Tuscany, as here cases are always interviewed by occupational health officials, based on agreements between these centres and Local Health Authorities. Indeed, differences in health and mental status between cases and controls are often obvious – even more so if we consider that interviews to respondents were relatively common among cases but not among population controls. We adjusted, therefore, by centre and type of interview (direct vs proxy) all our analyses. We also conducted a set of sensitivity analyses by leaving out each centre one at a time.

A second point is the larger proportion of proxy respondents among cases, associated with patients’ rapidly declining health. As proxies are expected to provide less detailed descriptions of the work and living environment, we adjusted our analyses by type of respondent. Residual confounding, if any, may have biased our findings towards the null.

Finally, we obtained a lower response rate among controls. We observed declining response rates among population controls in two studies conducted in the Casale Monferrato area in respectively, 1987–1993 and 2001–2006: responding controls dropped from 83 to 63% of eligible subjects [30, 31]. As response rate depends on education and socio-economic status, its difference between cases and controls may have biased our findings. Findings by Ferrante et al. [30] were confirmed by a re-analysis where education had been used as a proxy to socio-economic status [32]. We addressed the potential non-response bias by using ever employment as a blue-collar worker as a proxy: we first repeated our set of analyses by introducing it as a covariate and then by restricting analyses to blue collar workers. Both sets of results confirmed the OR increase by CE observed in our main analyses, even if the OR point estimates appeared to be slightly reduced and confidence intervals widened.

In conclusion, we conducted a population-based case–control study with nation-wide population basis. Having involved five centers serving regional populations ranging from Northern to Southern Italy, we included areas with historically different types and levels of industrial development. We took advantage of the ReNaM network and experience, in particular of its detailed questionnaire and, additionally, carried out a quantitative exposure assessment by linkage with SYN-JEM, a JEM developed using data (among the others) from Italy relative to a time span overlapping the work histories of cases and controls. We observed increased MM risk in the asbestos industry, manufacture of railroad equipment, ship building and repairing, chemical industry, oil refineries, iron and steel mills, glass production industry, constructions and freight transport by road and water, as well as in associated occupations. Non-occupational exposures to asbestos, as identified by the ReNaM categories for residential, familial and other non-occupational exposures, also entailed a remarkable increase in MM risk. Finally, we observed that MM risk was sub-linearly proportional to CE.

## Supplementary Information

**Additional file 1: Supplementary Table S1.** Characteristics of the study. **Supplementary Table S2.** Pleural mesothelioma in men and women: odds ratio (OR) and 95% confidence intervals (CI) by industry according to the International Standard Industry Classification (ISIC, 4-digit codes), 1971 – Industries with at least 3 exposed cases. **Supplementary Table S3.** Pleural mesothelioma in men and women: odds ratio (OR) and 95% confidence intervals (CI) by occupation, according to the International Standard Code of Occupations (ISCO, 3-digit codes), 1968 – Occupations with at least 3 exposed cases. **Supplementary Table S4.** Number of cases and controls, odds ratio (OR) and 95% confidence intervals (CI) by modality of exposure according to the Italian National Mesothelioma Register (ReNaM) classification. Reference category: unlikely/unknown exposures. All occupational exposure categories (definite, probable and possible) combined, lag 10 years. **Supplementary Table S5.** Number of cases and controls, odds ratio (OR) and 95% confidence intervals (CI) by modality of exposure according to the Italian National Mesothelioma Register (ReNaM) classification. Reference category: unlikely/unknown exposures. Distinct occupational categories (definite, probable and possible), lag 10 years. **Supplementary Table S6.** Number of cases and controls, odds ratio (OR) and 95% confidence intervals (CI) by modality of exposure according to the Italian National Mesothelioma Register (ReNaM) classification. Reference category: unlikely exposures, lag 10 years. **Supplementary Table S7.** Number of cases and controls, odds ratio (OR) and 95% confidence interval (CI) by exposure (ever vs never) and cumulative exposure from SYN-JEM, unlagged and at lag 10, 20 and 30 years, men. **Supplementary Table S8.** Number of cases and controls, odds ratio (OR) and 95% confidence intervals (CI) by modality of exposure, according to the Italian National Mesothelioma Register (ReNaM) classification. Reference category: unlikely/unknown exposure, lag 10 years. Analyses adjusted also by blue-collar status, as a proxy for socio-economic status. **Supplementary Table S9.** Number of cases and controls, odds ratio (OR) and 95% confidence interval (CI) by exposure (ever vs never) and cumulative exposure from SYN-JEM, unlagged and at lag 10, 20 and 30 years, men and women. Analyses adjusted also by blue-collar status, as a proxy for socio-economic status. **Supplementary Table S10.** Number of cases and controls, odds ratio (OR) and 95% confidence intervals (CI) by modality of exposure, according to the Italian National Mesothelioma Register (ReNaM) classification. Reference category: unlikely/unknown exposure, lag 10 years. Analyses restricted to blue-collar cases and controls. **Supplementary Table S11.** Number of cases and controls, odds ratio (OR) and 95% confidence interval (CI) by exposure (ever vs never) and cumulative exposure from SYN-JEM, unlagged and at lag 10, 20 and 30 years, men and women. Analyses restricted to blue-collar cases and controls.

## Data Availability

The datasets used and/or analysed during the current study are available from the corresponding author on reasonable request.

## Abbreviations

MISEM: Multicentre Italian Study on the Etiology of Mesothelioma
MM: Malignant mesothelioma
ReNaM: National Mesothelioma Registry
COR: Registry Operating Centre
ISIC: International Standard Industrial Classification of Economic Activities
ISCO: International Standard Classification of Occupations
IARC: International Agency for Research on Cancer
CE: cumulative exposure
OR: Odds Ratios
CI: Confidence Intervals
AIC: Akaike information criterion
BC: Blue-collar
