# Supplementary material for: Pleural mesothelioma risk by industry and occupation: results from the Multicentre Italian Study on the Etiology of Mesothelioma (MISEM)
Source: Environ Health. 2022 Jun 18;21:60. doi: 10.1186/s12940-022-00869-5 (PMC9206310; doi:10.1186/s12940-022-00869-5)
Supplement: Supplementary file 1 — Additional file 1: Supplementary Table S1. Characteristics of the study. Supplementary Table S2. Pleural mesothelioma in men and women: odds ratio (OR) and 95% confidence intervals (CI) by industry according to the International Standard Industry Classification (ISIC, 4-digit codes), 1971 – Industries with at least 3 exposed cases. Supplementary Table S3. Pleural mesothelioma in men and women: odds ratio (OR) and 95% confidence intervals (CI) by occupation, according to the International Standard Code of Occupations (ISCO, 3-digit codes), 1968 – Occupations with at least 3 exposed cases. Supplementary Table S4. Number of cases and controls, odds ratio (OR) and 95% confidence intervals (CI) by modality of exposure according to the Italian National Mesothelioma Register (ReNaM) classification. Reference category: unlikely/unknown exposures. All occupational exposure categories (definite, probable and possible) combined, lag 10 years. Supplementary Table S5. Number of cases and controls, odds ratio (OR) and 95% confidence intervals (CI) by modality of exposure according to the Italian National Mesothelioma Register (ReNaM) classification. Reference category: unlikely/unknown exposures. Distinct occupational categories (definite, probable and possible), lag 10 years. Supplementary Table S6. Number of cases and controls, odds ratio (OR) and 95% confidence intervals (CI) by modality of exposure according to the Italian National Mesothelioma Register (ReNaM) classification. Reference category: unlikely exposures, lag 10 years. Supplementary Table S7. Number of cases and controls, odds ratio (OR) and 95% confidence interval (CI) by exposure (ever vs never) and cumulative exposure from SYN-JEM, unlagged and at lag 10, 20 and 30 years, men. Supplementary Table S8. Number of cases and controls, odds ratio (OR) and 95% confidence intervals (CI) by modality of exposure, according to the Italian National Mesothelioma Register (ReNaM) classification. Reference category: unlikely/u [file 12940_2022_869_MOESM1_ESM.docx]

**Supplementary material**

Supplementary Table S1. Characteristics of the study

| ***Center*** | ***Population*** | ***Recruitment period*** | ***Cases*** | | ***Controls*** | |
| --- | --- | --- | --- | --- | --- | --- |
|  |  |  | ***Invited**** | ***Interviewed***  ***(%)*** | ***Invited*** | ***Interviewed***  ***(%)*** |
| Piedmont, province of Turin | 2 243 382 | 01/01/2012-31/12/2013 | 167 | 166  (99.4) | 392 | 167  (42.6) |
| Piedmont, Casale Monferrato | 84 200 | 01/01/2012-31/12/2013 | 68 | 68  (100) | 180 | 100  (55.6) |
| Lombardy | 9 973 397 | 01/01/2014-31/12/2014 | 211 | 205  (97.2) | 422 | 203  (48.1) |
| Veneto, Venice and Padua provinces | 1 181 751 | 01/01/2012-31/12/2013 | 75 | 74  (98.7) | 200 | 143  (71.5) |
| Tuscany | 3 667 780 | 01/01/2013-31/12/2013 | 85 | 79  (92.9) | 86 | 33  (38.4) |
| Apulia | 4 050 072 | 01/01/2012-31/12/2012 | 41 | 34  (82.9) | 112 | 72  (64.3) |
| Overall | 21 200 582 |  | 647 | 626  (96.8) | 1392 | 718  (51.6) |

* Eligible cases identified by each center through the rapid alert system during the recruitment period

Supplementary Table S2. Pleural mesothelioma in men and women: odds ratio (OR) and 95% confidence intervals (CI) by industry according to the International Standard Industry Classification (ISIC, 4-digit codes), 1971 – Industries with at least 3 exposed cases - the MISEM study, 2012-2015, Italy.

|  |  | **Men (953)** | | | **Women (391)** | | |
| --- | --- | --- | --- | --- | --- | --- | --- |
| **ISIC code*** | **Description** | **No. of exposed cases/controls** | **OR**** | **CI** | **No. of exposed cases/controls** | **OR**** | **CI** |
| 1110 | Agriculture and livestock production | 62/64 | 0.89 | (0.60; 1.33) | 20/22 | 1.63 | (0.82; 3.27) |
| 3111 | Slaughtering, preparing and preserving meat | 4/4 | 1.10 | (0.26; 4.63) | 0/2 | - | - |
| 3113 | Canning and preserving of fruits and vegetables | 0/1 | - | - | 3/0 | - | - |
| 3117 | Manufacture of bakery products | 8/4 | 2.10 | (0.60; 7.38) | 3/7 | 0.66 | (0.15; 2.80) |
| 3210 | Manufacture of textiles | 1/1 | 0.58 | (0.03; 9.61) | 5/2 | 3.42 | (0.61; 19.09) |
| 3211 | Spinning, weaving and finishing textiles | 12/8 | 1.40 | (0.55; 3.58) | 10/6 | 1.73 | (0.58; 5.13) |
| 3212 | Manufacture of made-up textile goods except wearing apparel | 1/6 | 0.13 | (0.01; 1.09) | 3/3 | 0.91 | (0.17; 4.94) |
| 3213 | Knitting mills | 3/2 | 1.91 | (0.30; 12.0) | 7/10 | 0.83 | (0.29; 2.39) |
| 3219 | Manufacture of textiles not elsewhere classified | 6/2 | 3.99 | (0.77; 20.8) | 3/2 | 2.51 | (0.40; 15.7) |
| 3220 | Manufacture of wearing apparel, except footwear | 5/2 | 2.56 | (0.46; 14.2) | 25/48 | 0.67 | (0.38; 1.18) |
| 3233 | Manufacture of products of leather and leather substitutes, except footwear and wearing apparel | 1/6 | 0.12 | (0.01; 1.02) | 4/2 | 2.38 | (0.39; 14.50) |
| 3240 | Manufacture of footwear, except vulcanized or moulded rubber or plastic footwear | 7/5 | 1.13 | (0.33; 3.89) | 2/7 | 0.23 | (0.04; 1.34) |
| 3300 | Manufacture of wood and wood products, including furniture | 7/4 | 1.74 | (0.50; 6.11) | - | - | - |
| 3311 | Sawmills, planing and other wood mills | 9/6 | 2.49 | (0.83; 7.47) | 0/0 | - | - |
| 3312 | Manufacture of wooden and cane containers and small cane ware | 4/5 | 0.74 | (0.19; 2.90) | 0/1 | - | - |
| 3320 | Manufacture of furniture and fixtures, except primarily of metal | 29/27 | 1.31 | (0.74; 2.30) | 3/1 | 6.62 | (0.64; 68.93) |
| 3411 | Manufacture of pulp, paper and paperboard | 11/3 | 3.33 | (0.91; 12.2) | 4/0 | - | - |
| 3419 | Manufacture of pulp, paper and paperboard articles not elsewhere classified | 5/4 | 1.41 | (0.35; 5.60) | 2/2 | 1.43 | (0.18; 11.03) |
| 3420 | Printing, publishing and allied industries | 11/17 | 0.69 | (0.31; 1.54) | 4/7 | 0.92 | (0.25; 3.40) |
| 3511 | Manufacture of basic industrial chemicals except fertilizers | 9/3 | 2.99 | (0.77; 11.7) | 1/1 | 0.77 | (0.05; 12.9) |
| 3513 | Manufacture of synthetic resins, plastic materials and man-made fibres except glass | 9/13 | 0.83 | (0.34; 2.03) | 4/3 | 1.89 | (0.40; 9.00) |
| 3521 | Manufacture of paints, varnishes and laquers | 3/2 | 2.19 | (0.35; 13.7) | 0/0 | - | - |
| 3522 | Manufacture of drugs and medicines | 6/4 | 1.56 | (0.42; 5.77) | 0/3 | - | - |
| 3523 | Manufacture of soap and cleaning preparations, perfumes, cosmetics and other toilet preparations | 4/5 | 1.09 | (0.28; 4.28) | 0/1 | - | - |
| 3530 | Petroleum refineries | 4/1 | 2.79 | (0.30; 25.9) | 1/0 | - | - |
| 3551 | Tyre and tube industries | 6/7 | 0.85 | (0.28; 2.63) | - | - | - |
| 3559 | Manufacture of rubber products not elsewhere classified | 9/7 | 1.21 | (0.43; 3.39) | 5/3 | 1.77 | (0.39; 8.02) |
| 3560 | Manufacture of plastic products not elsewhere classified | 8/7 | 1.31 | (0.45; 3.80) | 2/3 | 1.14 | (0.17; 7.59) |
| 3620 | Manufacture of glass and glass products | 11/6 | 2.32 | (0.80; 6.75) | 2/0 | - | - |
| 3690 | Manufacture of other non-metallic mineral products | 6/3 | 2.24 | (0.54; 9.31) | 0/0 | - | - |
| 3691 | Manufacture of structural clay products | 7/6 | 1.07 | (0.35; 3.29) | 3/1 | 3.25 | (0.30; 34.89) |
| 3692 | Manufacture of cement, lime and plaster | 6/2 | 2.94 | (0.57; 15.3) | 0/2 | - | - |
| 3699 | Manufacture of non-metallic mineral products not elsewhere classified | 17/5 | 3.43 | (1.23; 9.58) | 6/0 | - | - |
| 3710 | Iron and steel basic industries | 29/13 | 2.15 | (1.07; 4.29) | 2/2 | 1.56 | (0.20; 12.4) |
| 3720 | Non-ferrous metal basic industries | 7/6 | 1.18 | (0.39; 3.62) | 1/0 | - | - |
| 3811 | Manufacture of cutlery, hand tools and general hardware | 7/4 | 1.89 | (0.53; 6.73) | 1/0 | - | - |
| 3812 | Manufacture of furniture and fixtures primarily of metal | 6/3 | 2.52 | (0.58; 10.9) | 0/0 | - | - |
| 3813 | Manufacture of structural metal products | 54/40 | 1.47 | (0.93; 2.31) | 3/2 | 2.88 | (0.39; 21.31) |
| 3819 | Manufacture of fabricated metal products except machinery and equipment not elsewhere classified | 58/49 | 1.12 | (0.72; 1.74) | 8/8 | 2.19 | (0.71; 6.69) |
| 3822 | Manufacture of agricultural machinery and equipment | 4/7 | 0.90 | (0.25; 3.24) | - | - | - |
| 3823 | Manufacture of metal and wood working machinery | 3/4 | 1.35 | (0.29; 6.29) | 1/0 | - | - |
| 3824 | Manufacture of special industrial machinery and equipment except metal and wood working machinery | 57/36 | 1.87 | (1.19; 2.93) | 1/5 | 0.21 | (0.02; 1.88) |
| 3825 | Manufacture of office, computing and accounting machinery | 7/7 | 0.95 | (0.33; 2.78) | 0/1 | - | - |
| 3831 | Manufacture of electrical industrial machinery and apparatus | 8/11 | 0.65 | (0.25; 1.64) | 3/2 | 3.26 | (0.48; 22.19) |
| 3832 | Manufacture of radio, television and communication equipment and apparatus | 6/11 | 0.50 | (0.18; 1.39) | 4/4 | 1.88 | (0.43; 8.22) |
| 3833 | Manufacture of electrical appliances and housewares | 18/23 | 0.84 | (0.44; 1.61) | 3/5 | 0.81 | (0.18; 3.64) |
| 3839 | Manufacture of electrical apparatus and supplies not elsewhere classified | 11/11 | 1.11 | (0.46; 2.67) | 3/0 | - | - |
| 3841 | Ship building and repairing | 12/6 | 2.34 | (0.82; 6.70) | 1/0 | - | - |
| 3842 | Manufacture of railroad equipment | 23/3 | 8.07 | (2.35; 27.7) | 0/0 | - | - |
| 3843 | Manufacture of motor vehicles | 46/34 | 1.14 | (0.68; 1.89) | 3/7 | 0.61 | (0.15; 2.54) |
| 3844 | Manufacture of motorcycles and bicycles | 4/5 | 0.98 | (0.25; 3.76) | 1/1 | 0.90 | (0.05; 15.21) |
| 3845 | Manufacture of aircraft | 3/2 | 1.36 | (0.21; 8.63) | - | - | - |
| 3901 | Manufacture of jewellery and related articles | 4/10 | 0.36 | (0.11; 1.23) | 0/6 | - | - |
| 3909 | Manufacturing industries not elsewhere classified | 28/17 | 1.87 | (0.97; 3.61) | 1/6 | 0.21 | (0.02; 1.81) |
| 4101 | Electric light and power | 8/15 | 0.53 | (0.21; 1.30) | 1/1 | 0.82 | (0.05; 14.15) |
| 4200 | Water works and supply | 5/0 | - | - | - | - | - |
| 5000 | Construction | 119/77 | 1.94 | (1.39; 2.71) | 3/3 | 1.81 | (0.32; 10.3) |
| 6100 | Wholesale trade | 31/39 | 0.84 | (0.50; 1.39) | 4/12 | 0.52 | (0.16; 1.74) |
| 6200 | Retail trade | 39/79 | 0.50 | (0.33; 0.76) | 16/38 | 0.55 | (0.28; 1.06) |
| 6310 | Restaurants, cafes and other eating and drinking places | 5/9 | 0.73 | (0.23; 2.37) | 0/7 | - | - |
| 6320 | Hotels, rooming houses, camps and other lodging places | 5/18 | 0.34 | (0.12; 0.96) | 5/13 | 0.64 | (0.21; 1.93) |
| 7111 | Railway transport | 7/10 | 0.94 | (0.34; 2.59) | - | - | - |
| 7112 | Urban, suburban and inter-urban highway passenger transport | 6/7 | 0.86 | (0.27; 2.73) | - | - | - |
| 7114 | Freight transport by road | 21/9 | 2.75 | (1.21; 6.28) | 2/0 | - | - |
| 7116 | Supporting services to land transport | 4/8 | 0.62 | (0.18; 2.10) | - | - | - |
| 7121 | Ocean and coastal water transport | 7/5 | 1.51 | (0.44; 5.22) | 0/1 | - | - |
| 7123 | Supporting services to water transport | 8/2 | 4.78 | (0.92; 24.8) | 1/0 | - | - |
| 7200 | Communication | 9/12 | 0.87 | (0.36; 2.14) | 2/2 | 0.79 | (0.08; 7.72) |
| 8101 | Monetary institutions | 13/23 | 0.51 | (0.25; 1.04) | 0/5 | - | - |
| 8200 | Insurance | 2/10 | 0.20 | (0.04; 0.96) | 3/5 | 0.76 | (0.16; 3.66) |
| 8321 | Legal services | 1/5 | 0.15 | (0.02; 1.28) | 4/5 | 1.19 | (0.29; 4.79) |
| 8323 | Data processing and tabulating services | 4/3 | 1.81 | (0.37; 8.74) | 0/1 | - | - |
| 8324 | Engineering, architectural and technical services | 5/20 | 0.28 | (0.10; 0.77) | 3/3 | 1.65 | (0.30; 9.12) |
| 8329 | Business services, except machinery and equipment rental and leasing, not elsewhere classified | 11/9 | 1.88 | (0.74; 4.79) | 10/3 | 5.17 | (1.36; 19.8) |
| 9100 | Public administration and defence | 15/31 | 0.49 | (0.26; 0.96) | 2/6 | 0.70 | (0.13; 3.76) |
| 9310 | Education services | 14/37 | 0.41 | (0.22; 0.79) | 17/31 | 0.76 | (0.39; 1.48) |
| 9331 | Medical, dental and other health services | 12/27 | 0.56 | (0.27; 1.16) | 4/20 | 0.34 | (0.11; 1.05) |
| 9340 | Welfare institutions | 1/5 | 0.33 | (0.04; 2.91) | 3/4 | 1.10 | (0.23; 5.30) |
| 9490 | Amusement and recreational services not elsewhere classified | 3/6 | 0.55 | (0.12; 2.39) | 2/1 | 3.84 | (0.26; 57.71) |
| 9513 | Repair of motor vehicles and motorcycles | 14/22 | 0.55 | (0.26; 1.13) | 2/0 | - | - |
| 9519 | Other repair shops not elsewhere classified | 3/2 | 1.03 | (0.16; 6.81) | - | - | - |
| 9520 | Laundries, laundry services, and cleaning and dyeing plants | 1/0 | - | - | 3/2 | 2.03 | (0.28; 14.57) |
| 9530 | Domestic services | 5/3 | 2.20 | (0.51; 9.54) | 17/24 | 0.92 | (0.46; 1.84) |
| 9591 | Barber and beauty shops | 1/1 | 0.74 | (0.04; 14.01) | 5/3 | 3.06 | (0.64; 14.60) |
| 9599 | Personal services not elsewhere classified | 5/8 | 0.72 | (0.23; 2.31) | 3/5 | 0.64 | (0.14; 2.99) |
|  |  |  |  |  |  |  |  |
|  | Undefined or inadequately defined economic activity | 19/19 | 1.37 | (0.68; 2.74) | 6/7 | 1.96 | (0.58; 6.61) |
|  | Military service | 108/157 | 0.65 | (0.47; 0.91) | 1/0 | - | - |
|  | Unemployment | 3/5 | 0.70 | (0.16; 3.11) | 10/18 | 0.86 | (0.36; 2.05) |
|  | Never seeking employment | - | - | - | 4/5 | 1.66 | (0.31; 8.87) |

* Industries with at least 3 exposed cases; ** OR adjusted by centre and age. Nec: not elsewhere classified

Supplementary Table S3. Pleural mesothelioma in men and women: odds ratio (OR) and 95% confidence intervals (CI) by occupation, according to the International Standard Code of Occupations (ISCO, 3-digit codes), 1968 – Occupations with at least 3 exposed cases – the MISEM study, 2012-2015, Italy.

|  |  | **Men (953)** | | | **Women (391)** | | |
| --- | --- | --- | --- | --- | --- | --- | --- |
| **ISCO Code*** | **Description** | **No. of exposed cases/controls** | **OR**** | **CI** | **No. of exposed cases/controls** | **OR**** | **CI** |
| 011 | chemist | 3/2 | 1.65 | (0.26; 10.4) | - | - | - |
| 024 | mechanical engineers | 3/3 | 0.93 | (0.18; 4.74) | - | - | - |
| 031 | surveyors | 4/4 | 1.06 | (0.25; 4.56) | - | - | - |
| 032 | draughtsmen | 11/18 | 0.68 | (0.31; 1.49) | 1/0 | - | - |
| 033 | civil engineering technicians | 3/4 | 0.99 | (0.21; 4.69) | - | - | - |
| 034 | electrical and electronics engineering technicians | 4/1 | 4.90 | (0.53; 45.2) | 0/0 | - | - |
| 035 | mechanical engineering technician | 4/1 | 3.55 | (0.38; 33.3) | 0/0 | - | - |
| 061 | medical doctors | 3/5 | 0.85 | (0.20; 3.67) | - | - | - |
| 071 | professional nurses | 1/6 | 0.21 | (0.02; 1.91) | 4/7 | 1.01 | (0.28; 3.71) |
| 072 | nursing personnel nec | 0/3 | - | - | 3/6 | 0.90 | (0.21; 3.95) |
| 090 | economists | 3/1 | 3.13 | (0.31; 31.4) | 0/0 | - | - |
| 110 | accountants | 4/10 | 0.34 | (0.10; 1.12) | 1/11 | 0.10 | (0.01; 0.83) |
| 131 | university and higher education teachers | 3/9 | 0.37 | (0.10; 1.40) | 1/4 | 0.33 | (0.03; 3.23) |
| 132 | secondary education teachers | 4/19 | 0.22 | (0.07; 0.66) | 5/11 | 0.61 | (0.20; 1.90) |
| 133 | primary education teachers | 1/2 | 0.74 | (0.06; 8.72) | 5/4 | 1.25 | (0.30; 5.30) |
| 180 | athletes, sportsmen and related workers | 3/1 | 4.48 | (0.41; 48.5) | 0/0 | - | - |
| 211 | general managers | 11/12 | 1.04 | (0.45; 2.44) | - | - | - |
| 212 | production managers (except farm) | 3/3 | 1.57 | (0.30; 8.14) | - | - | - |
| 219 | managers nec | 8/17 | 0.47 | (0.19; 1.12) | 1/3 | 0.41 | (0.04; 4.45) |
| 331 | bookkeepers and cashiers | 9/20 | 0.45 | (0.20; 1.03) | 2/9 | 0.33 | (0.07; 1.61) |
| 370 | mail distribution clerks | 11/14 | 0.78 | (0.34; 1.78) | 1/1 | 1.71 | (0.06; 51.1) |
| 390 | clerical and related workers nec | 3/14 | 0.18 | (0.05; 0.66) | 8/9 | 0.91 | (0.31; 2.66) |
| 391 | stock clerks | 35/16 | 2.49 | (1.33; 4.66) | 2/3 | 0.83 | (0.13; 5.28) |
| 392 | material and production planning clerks | 3/6 | 0.64 | (0.15; 2.70) | 0/1 | - | - |
| 393 | correspondence and reporting clerks | 35/66 | 0.58 | (0.36; 0.91) | 21/43 | 0.79 | (0.42; 1.50) |
| 410 | working proprietors (wholesale and retail trade) | 8/29 | 0.27 | (0.12; 0.60) | 6/6 | 2.13 | (0.62; 7.25) |
| 431 | technical salesmen and service advisers | 4/5 | 0.85 | (0.22; 3.33) | 0/1 | - | - |
| 432 | commercial travellers and manufacturers' agents | 14/29 | 0.58 | (0.30; 1.14) | - | - | - |
| 451 | salesmen, shop assistants and demonstrators | 18/39 | 0.46 | (0.25; 0.84) | 12/29 | 0.49 | (0.23; 1.04) |
| 452 | street vendors, canvassers and newsvendors | 4/2 | 3.65 | (0.64; 20.7) | 0/1 | - | - |
| 532 | waiters bartenders and related workers | 8/14 | 0.73 | (0.28; 1.87) | 2/10 | 0.31 | (0.06; 1.51) |
| 540 | maids and related housekeeping service workers nec | 1/3 | 0.47 | (0.05; 4.79) | 17/25 | 0.91 | (0.46; 1.80) |
| 551 | building caretakers | 6/2 | 3.57 | (0.69; 18.4) | 1/5 | 0.24 | (0.03; 2.26) |
| 552 | charworkers, cleaners and related workers | 6/10 | 0.60 | (0.20; 1.76) | 10/2 | 8.16 | (1.68; 39.7) |
| 560 | launderers, dry-cleaners and pressers | 2/0 | - | - | 4/5 | 1.00 | (0.24; 4.20) |
| 570 | hairdressers, barbers, beauticians and related workers | 1/1 | 0.74 | (0.04; 14.0) | 4/2 | 2.46 | (0.42; 14.3) |
| 581 | fire-fighters | 4/1 | 4.73 | (0.50; 44.4) | 0/0 | - | - |
| 582 | policemen and detectives | 7/12 | 0.75 | (0.28; 2.00) | - | - | - |
| 589 | protective service workers nec | 27/72 | 0.51 | (0.28; 0.93) | - | - | - |
| 599 | other service workers | 6/2 | 4.38 | (0.86; 22.3) | 1/0 | - | - |
| 611 | general farmers | 6/15 | 0.67 | (0.24; 1.84) | 0/4 | - | - |
| 621 | general farm workers | 33/38 | 0.65 | (0.38; 1.11) | 13/13 | 1.85 | (0.78; 4.41) |
| 622 | field crop and vegetable farm workers | 6/0 | - | - | 5/2 | 3.91 | (0.71; 21.6) |
| 623 | orchard, vineyard and related tree and shrub crop workers | 9/6 | 1.47 | (0.50; 4.32) | 4/2 | 3.12 | (0.53; 18.5) |
| 624 | livestock workers | 5/6 | 0.72 | (0.21; 2.47) | 1/0 | - | - |
| 700 | production supervisors and general foremen | 12/14 | 0.85 | (0.37; 1.94) | - | - | - |
| 711 | miners and quarrymen | 5/2 | 1.87 | (0.32; 11.1) | - | - | - |
| 721 | metal smelting, converting and refining furnace men | 14/6 | 2.45 | (0.91; 6.60) | 0/0 | - | - |
| 722 | metal rolling-mill workers | 3/1 | 2.57 | (0.25; 25.9) | 0/0 | - | - |
| 724 | metal casters | 7/1 | 5.74 | (0.67; 49.0) | 0/0 | - | - |
| 729 | metal processors nec^+^ | 3/1 | 4.97 | (0.48; 51.4) | 1/0 | - | - |
| 734 | paper makers | 3/1 | 3.18 | (0.32; 31.7) | 0/0 | - | - |
| 749 | chemical processors and related workers nec | 4/0 | - | - | 2/1 | 2.87 | (0.24; 33.9) |
| 752 | spinners and winders | 5/3 | 1.56 | (0.36; 6.75) | 5/2 | 2.88 | (0.51; 16.4) |
| 754 | weavers and related workers | 3/1 | 2.25 | (0.22; 22.98) | 10/6 | 2.15 | (0.72; 6.40) |
| 755 | knitters | 1/0 | - | - | 7/10 | 0.84 | (0.30; 2.37) |
| 773 | butchers and meat preparers | 3/5 | 0.74 | (0.17; 3.23) | - | - | - |
| 776 | bakers, pastrycooks and confectionery makers | 7/5 | 1.28 | (0.39; 4.23) | 2/5 | 0.58 | (0.10; 3.34) |
| 791 | tailors and dressmakers | 0/2 | - | - | 19/33 | 0.78 | (0.41; 1.46) |
| 795 | sewers and embroiderers | 1/1 | 0.88 | (0.05; 15.5) | 8/11 | 0.85 | (0.31; 2.32) |
| 796 | upholsterers and related workers | 6/5 | 1.51 | (0.43; 5.26) | - | - | - |
| 801 | shoemakers and shoe repairers | 3/4 | 0.89 | (0.18; 4.36) | - | - | - |
| 802 | shoe cutters, lasters, sewers and related workers | 2/1 | 2.40 | (0.21; 28.0) | 3/7 | 0.44 | (0.10; 1.87) |
| 803 | leather good makers | 0/2 | - | - | 3/3 | 0.83 | (0.15; 4.51) |
| 810 | cabinetmakers and related woodworkers | 4/7 | 0.64 | (0.18; 2.26) | - | - | - |
| 811 | cabinetmakers | 9/9 | 1.12 | (0.43; 2.94) | - | - | - |
| 819 | cabinetmakers and related woodworkers nec | 4/3 | 1.15 | (0.25; 5.32) | 1/1 | 0.91 | (0.05; 15.2) |
| 820 | stone cutters and carvers | 3/1 | 3.59 | (0.35; 36.4) | 0/0 | - | - |
| 831 | blacksmiths, hammersmiths and forging-press operators | 15/19 | 0.89 | (0.43; 1.81) | 0/1 | - | - |
| 833 | machine-tool setter-operators | 14/24 | 0.53 | (0.27; 1.06) | 1/0 | - | - |
| 834 | machine-tool operators | 31/20 | 1.64 | (0.90; 2.99) | 1/0 | - | - |
| 839 | blacksmiths, toolmakers and machine-tool operators nec | 17/2 | 10.38 | (2.34; 46.0) | 3/0 | - | - |
| 841 | machinery fitters and machine assemblers | 15/17 | 0.86 | (0.41; 1.78) | 1/1 | 2.37 | (0.14; 40.9) |
| 843 | motor-vehicle mechanics | 8/15 | 0.52 | (0.21; 1.30) | - | - | - |
| 849 | machinery fitters, machine assemblers and precision instrument makers (except electrical) nec | 46/31 | 1.51 | (0.92; 2.47) | 0/1 | - | - |
| 851 | electrical fitters | 5/2 | 3.05 | (0.57; 16.3) | 0/0 | - | - |
| 853 | electrical and electronic equipment assemblers | 3/5 | 0.48 | (0.11; 2.08) | 1/1 | 1.45 | (0.08; 25.9) |
| 854 | radio and television repairmen | 4/1 | 4.27 | (0.43; 42.7) | 0/0 | - | - |
| 855 | electrical wiremen | 28/19 | 1.80 | (0.97; 3.34) | - | - | - |
| 857 | electric linemen and cable jointers | 5/5 | 0.90 | (0.25; 3.20) | 0/1 | - | - |
| 859 | electrical fitters and related electrical and electronics workers nec | 7/8 | 0.92 | (0.33; 2.61) | 0/2 | - | - |
| 871 | plumbers and pipe fitters | 26/12 | 2.47 | (1.20; 5.11) | 0/0 | - | - |
| 872 | welders and flame-cutters | 45/13 | 3.97 | (2.06; 7.64) | 2/2 | 2.11 | (0.26; 16.9) |
| 873 | sheet-metal workers | 15/4 | 3.79 | (1.20; 11.9) | 0/0 | - | - |
| 874 | structural metal preparers and erectors | 23/14 | 2.11 | (1.02; 4.35) | - | - | - |
| 880 | jewellery and precious metal workers | 4/7 | 0.50 | (0.13; 1.86) | 1/4 | 0.41 | (0.04; 4.19) |
| 891 | glass formers, cutters, grinders and finishers | 12/3 | 3.95 | (1.01; 15.4) | 0/0 | - | - |
| 901 | rubber and plastic product makers (except tire makers and tire vulcanisers) | 9/7 | 1.32 | (0.47; 3.73) | 3/3 | 1.65 | (0.31; 8.86) |
| 931 | painters, construction | 10/5 | 2.25 | (0.72; 7.00) | 0/0 | - | - |
| 939 | painters nec | 4/4 | 1.09 | (0.26; 4.60) | 1/0 | - | - |
| 943 | non-metallic mineral product makers | 9/4 | 1.99 | (0.60; 6.68) | 4/1 | 5.02 | (0.53; 47.6) |
| 951 | bricklayers, stonemasons and tile setters | 21/19 | 0.80 | (0.39; 1.62) | - | - | - |
| 953 | roofers | 6/1 | 6.51 | (0.77; 55.1) | 0/0 | - | - |
| 954 | carpenters, joiners and parquetry workers | 14/14 | 1.33 | (0.58; 3.02) | 1/0 | - | - |
| 956 | insulators | 5/4 | 1.41 | (0.35; 5.75) | - | - | - |
| 959 | construction workers nec | 58/28 | 2.59 | (1.59; 4.23) | 0/0 | - | - |
| 969 | stationary engine and related equipment operators nec | 6/5 | 1.29 | (0.37; 4.42) | - | - | - |
| 971 | dockers and freight handlers | 12/14 | 0.99 | (0.44; 2.25) | 7/6 | 1.43 | (0.46; 4.50) |
| 973 | crane and hoist operators | 4/3 | 1.25 | (0.26; 6.05) | - | - | - |
| 974 | earth-moving and related machinery operators | 4/11 | 0.41 | (0.12; 1.33) | - | - | - |
| 982 | ships' engine-room ratings | 6/1 | 7.38 | (0.84; 65.0) | 0/0 | - | - |
| 983 | railway engine-drivers and firemen | 4/2 | 3.01 | (0.49; 18.4) | 0/0 | - | - |
| 985 | motor-vehicle drivers | 29/28 | 1.12 | (0.64; 1.96) | - | - | - |
| 999 | labourers nec | 67/54 | 1.30 | (0.84; 1.99) | 26/20 | 2.68 | (1.34; 5.38) |
|  |  |  |  |  |  |  |  |
|  | other occupational conditions with no corresponding ISCO code, including military service and unemployment | 18/9 | 3.02 | (1.28; 7.10) | 3/7 | 0.92 | (0.20; 4.12) |
|  | undefined or inadequately defined occupations | 76/88 | 0.62 | (0.41; 0.94) | 15/23 | 1.07 | (0.50; 2.29) |

* Occupations with at least 3 cases exposed; ** OR adjusted for centre and age. Nec: not elsewhere classified

Supplementary Table S4 – Number of cases and controls, odds ratio (OR) and 95% confidence intervals (CI) by modality of exposure according to the Italian National Mesothelioma Register (ReNaM) classification. Reference category: unlikely/unknown exposures. All occupational exposure categories (definite, probable and possible) combined, lag 10 years – the MISEM study, 2012-2015, Italy.

|  | **Men and women** | | | | **Men** | | | | **Women** | | | |
| --- | --- | --- | --- | --- | --- | --- | --- | --- | --- | --- | --- | --- |
| **Exposure** | **Cases** | **Ctrls** | **OR*** | **CI** | **Cases** | **Ctrls** | **OR**** | **CI** | **Cases** | **Ctrls** | **OR**** | **CI** |
| Unlikely/unknown exposure | 122 | 382 | 1.00 | (ref) | 67 | 248 | 1.00 | (ref) | 55 | 134 | 1.00 | (ref) |
| Occupational | 404 | 190 | 9.37 | (6.77-13.0) | 354 | 163 | 10.0 | (6.90-14.5) | 50 | 27 | 6.28 | (3.03-13.0) |
| Familial | 40 | 45 | 4.85 | (2.81-8.39) | 14 | 28 | 2.83 | (1.29-6.20) | 26 | 17 | 10.50 | (4.16-26.4) |
| Residential | 44 | 75 | 2.46 | (1.47-4.13) | 20 | 42 | 2.21 | (1.11-4.38) | 24 | 33 | 3.22 | (1.33-7.80) |
| Other non-occupational | 13 | 23 | 2.62 | (1.19-5.76) | 7 | 8 | 4.62 | (1.49-14.3) | 6 | 15 | 2.12 | (0.63-7.15) |
|  |  |  |  |  |  |  |  |  |  |  |  |  |
| *P-Wald test gender interaction* |  |  | 0.07 |  |  |  |  |  |  |  |  |  |
| AIC |  |  | 1458.62 |  |  |  | 1051.17 |  |  |  | 416.73 |  |

* OR adjusted by gender, centre, age and type of interview; ** OR adjusted by centre, age and type of interview. AIC: Akaike information criterion

Supplementary Table S5 – Number of cases and controls, odds ratio (OR) and 95% confidence intervals (CI) by modality of exposure according to the Italian National Mesothelioma Register (ReNaM) classification. Reference category: unlikely/unknown exposures. Distinct occupational categories (definite, probable and possible), lag 10 years – the MISEM study, 2012-2015, Italy.

|  | **Men and women** | | | | **Men** | | | | **Women** | | | |
| --- | --- | --- | --- | --- | --- | --- | --- | --- | --- | --- | --- | --- |
| **Exposure** | **Cases** | **Ctrls** | **OR*** | **CI** | **Cases** | **Ctrls** | **OR**** | **CI** | **Cases** | **Ctrls** | **OR**** | **CI** |
| Unlikely/unknown exposure | 122 | 382 | 1.00 | (ref) | 67 | 248 | 1.00 | (ref) | 55 | 134 | 1.00 | (ref) |
| Occupational, definite | 315 | 99 | 15.6 | (10.7-22.6) | 283 | 88 | 16.2 | (10.7-24.6) | 32 | 11 | 11.6 | (4.40-30.8) |
| Occupational, probable | 28 | 11 | 9.60 | (4.35-21.2) | 23 | 8 | 12.7 | (5.07-31.6) | 5 | 3 | 2.77 | (0.50-15.4) |
| Occupational, possible | 61 | 80 | 3.03 | (1.93-4.77) | 48 | 67 | 2.82 | (1.68-4.72) | 13 | 13 | 3.88 | (1.39-10.8) |
| Familial | 40 | 45 | 4.68 | (2.69-8.11) | 14 | 28 | 2.55 | (1.16-5.63) | 26 | 17 | 11.0 | (4.31-28.2) |
| Residential | 44 | 75 | 2.42 | (1.44-4.09) | 20 | 42 | 2.16 | (1.08-4.31) | 24 | 33 | 3.41 | (1.39-8.37) |
| Other non-occupational | 13 | 23 | 2.62 | (1.18-5.79) | 7 | 8 | 4.70 | (1.50-14.8) | 6 | 15 | 2.22 | (0.65-7.53) |
|  |  |  |  |  |  |  |  |  |  |  |  |  |
| *P-Wald test gender interaction* |  |  | 0.10 |  |  |  |  |  |  |  |  |  |
| AIC |  |  | 1410.36 |  |  |  | 1004.58 |  |  |  | 416.52 |  |

* OR adjusted by gender, centre, age and type of interview; ** OR adjusted by centre, age and type of interview. AIC: Akaike information criterion

Supplementary Table S6 - Number of cases and controls, odds ratio (OR) and 95% confidence intervals (CI) by modality of exposure according to the Italian National Mesothelioma Register (ReNaM) classification. Reference category: unlikely exposures, lag 10 years – the MISEM study, 2012-2015, Italy

|  | **Men and women** | | | | **Men** | | | | **Women** | | | |
| --- | --- | --- | --- | --- | --- | --- | --- | --- | --- | --- | --- | --- |
| **Exposure** | **Cases** | **Ctrls** | **OR*** | **CI** | **Cases** | **Ctrls** | **OR**** | **CI** | **Cases** | **Ctrls** | **OR**** | **CI** |
| Unlikely exposure | 44 | 228 | 1.00 | (ref) | 26 | 147 | 1.00 | (ref) | 18 | 81 | 1.00 | (ref) |
| Occupational, definite or probable | 343 | 110 | 19.6 | (12.5-30.8) | 306 | 96 | 22.5 | (13.1-38.8) | 37 | 14 | 9.73 | (3.63-26.1) |
| Occupational, possible | 61 | 80 | 3.93 | (2.35-6.59) | 48 | 67 | 3.88 | (2.11-7.14) | 13 | 13 | 4.05 | (1.36-12.1) |
| Familial | 40 | 45 | 5.83 | (3.22-10.6) | 14 | 28 | 3.37 | (1.45-7.85) | 26 | 17 | 11.1 | (4.15-29.8) |
| Residential | 44 | 75 | 3.05 | (1.72-5.42) | 20 | 42 | 2.90 | (1.36-6.20) | 24 | 33 | 3.50 | (1.34-9.10) |
| Other non-occupational | 13 | 23 | 3.40 | (1.48-7.84) | 7 | 8 | 6.62 | (2.00-21.9) | 6 | 15 | 2.37 | (0.66-8.60) |
| Unknown | 78 | 154 | 1.77 | (1.06-2.95) | 41 | 101 | 2.00 | (1.05-3.81) | 37 | 53 | 1.23 | (0.49-3.07) |
|  |  |  |  |  |  |  |  |  |  |  |  |  |
| *P-Wald test gender interaction* |  |  | *0.044* |  |  |  |  |  |  |  |  |  |
| AIC |  |  | 1406.80 |  |  |  | 1000.35 |  |  |  | 418.43 |  |

* OR adjusted by gender, centre, age and type of interview; ** OR adjusted by centre, age and type of interview. AIC: Akaike information criterion

Supplementary Table S7 – Number of cases and controls, odds ratio (OR) and 95% confidence interval (CI) by exposure (ever vs never) and cumulative exposure from SYN-JEM, unlagged and at lag 10, 20 and 30 years, men – the MISEM study, 2012-2015, Italy

|  |  |  | **Lag 0** | | **Lag 10** | | **Lag 20** | | **Lag 30** | |
| --- | --- | --- | --- | --- | --- | --- | --- | --- | --- | --- |
| **SYN-JEM exposure index** | **Cases** | **Ctrls** | **OR*** | **CI** | **OR*** | **CI** | **OR*** | **CI** | **OR*** | **CI** |
| **ever/never exposed** |  |  |  |  |  |  |  |  |  |  |
| Unexposed | 154 | 253 | 1.00 | (ref) | 1.00 | (ref) | 1.00 | (ref) | 1.00 | (ref) |
| Exposed | 302 | 232 | 2.28 | (1.69-3.07) | 2.26 | (1.68-3.05) | 2.30 | (1.71-3.09) | 2.11 | (1.58-2.84) |
| AIC |  |  | 1183.93 |  | 1182.69 |  | 1182.16 |  |  | 1195.36 |
| **below/above median cumulative exposure (in f/mL-y)** | | | | | | | | | | |
| Unexposed | 154 | 253 | 1.00 | (ref) | 1.00 | (ref) | 1.00 | (ref) | 1.00 | (ref) |
| < 0.77 | 106 | 116 | 1.73 | (1.19-2.52) | 1.68 | (1.15-2.45) | 1.74 | (1.19-2.53) | 1.61 | (1.11-2.33) |
| ≥ 0.77 | 196 | 116 | 2.74 | (1.95-3.83) | 2.75 | (1.97-3.85) | 2.78 | (1.98-3.89) | 2.58 | (1.84-3.62) |
| *P-trend* |  |  | *< 0.001* |  | *< 0.001* |  | *< 0.001* |  | *< 0.001* |  |
| AIC |  |  | 1180.59 |  | 1178.54 |  | 1178.67 |  | 1191.80 |  |
| **tertiles of cumulative exposure (in f/mL-y)** | | | | | | | | | | |
| Unexposed | 154 | 253 | 1.00 | (ref) | 1.00 | (ref) | 1.00 | (ref) | 1.00 | (ref) |
| <0.32 | 59 | 78 | 1.32 | (0.85-2.07) | 1.36 | (0.87-2.13) | 1.49 | (0.96-2.32) | 1.20 | (0.77-1.87) |
| 0.32-1.49 | 91 | 76 | 2.36 | (1.58-3.51) | 2.21 | (1.48-3.30) | 2.20 | (1.47-3.29) | 2.13 | (1.43-3.16) |
| >1.49 | 152 | 78 | 2.97 | (2.04-4.32) | 3.02 | (2.08-4.39) | 3.04 | (2.09-4.43) | 2.92 | (2.00-4.27) |
| *P-trend* |  |  | *< 0.001* |  | *< 0.001* |  | *< 0.001* |  | *< 0.001* |  |
| AIC |  |  | 1176.89 |  | 1176.24 |  | 1177.75 |  | 1186.76 |  |
| **cumulative exposure, continuos** | | | | | | | | | | |
| Unexposed | 154 | 253 | 1.00 | (ref) | 1.00 | (ref) | 1.00 | (ref) | 1.00 | (ref) |
| Unit exposure: 1 f/mL-y | 302 | 232 | 1.27 | (1.15-1.41) | 1.27 | (1.14-1.41) | 1.27 | (1.15-1.41) | 1.30 | (1.16-1.46) |
| AIC |  |  | 1191.08 |  | 1189.80 |  | 1190.53 |  | 1197.01 |  |
| **log-cumulative exposure, continuos** | | | | | | | | | | |
| Unexposed | 154 | 253 | 1.00 | (ref) | 1.00 | (ref) | 1.00 | (ref) | 1.00 | (ref) |
| Unit exposure : 1 log(f/mL-y+1) | 302 | 232 | 2.05 | (1.58-2.64) | 2.05 | (1.58-2.64) | 2.05 | (1.59-2.65) | 2.12 | (1.64-2.79) |
| AIC |  |  | 1182.14 |  | 1180.76 |  | 1181.57 |  | 1188.61 |  |

* OR adjusted by centre, gender, age and type of interview. AIC: Akaike information criterion

Supplementary Table S8 – Number of cases and controls, odds ratio (OR) and 95% confidence intervals (CI) by modality of exposure, according to the Italian National Mesothelioma Register (ReNaM) classification. Reference category: unlikely/unknown exposure, lag 10 years – the MISEM study, 2012-2015, Italy. Analyses adjusted also by blue-collar status, as a proxy for socio-economic status.

|  | **Men and women** | | | | **Men** | | | | **Women** | | | |
| --- | --- | --- | --- | --- | --- | --- | --- | --- | --- | --- | --- | --- |
| **Exposure** | **Cases** | **Ctrls** | **OR*** | **CI** | **Cases** | **Ctrls** | **OR**** | **CI** | **Cases** | **Ctrls** | **OR**** | **CI** |
| Unlikely/unknown exposure | 122 | 382 | 1.00 | (ref) | 67 | 248 | 1.00 | (ref) | 55 | 134 | 1.00 | (ref) |
| Occupational, definite or probable | 343 | 110 | 14.4 | (9.85-20.9) | 306 | 96 | 15.3 | (9.94-23.4) | 37 | 14 | 8.99 | (3.67-22.0) |
| Occupational, possible | 61 | 80 | 2.92 | (1.83-4.64) | 48 | 67 | 2.69 | (1.58-4.60) | 13 | 13 | 3.82 | (1.34-11.0) |
| Familial | 40 | 45 | 4.41 | (2.52-7.69) | 14 | 28 | 2.50 | (1.13-5.55) | 26 | 17 | 9.83 | (3.82-25.3) |
| Residential | 44 | 75 | 2.48 | (1.47-4.19) | 20 | 42 | 2.31 | (1.15-4.63) | 24 | 33 | 3.19 | (1.31-7.75) |
| Other non-occupational | 13 | 23 | 2.57 | (1.16-5.72) | 7 | 8 | 4.58 | (1.45-14.4) | 6 | 15 | 2.17 | (0.64-7.35) |
| *P-Wald test gender interaction* |  |  | *0.06* |  |  |  |  |  |  |  |  |  |
| AIC |  |  | 1406.23 |  |  |  | 1001.79 |  |  |  | 417.24 |  |

* OR adjusted by centre, gender, age, and type of interview; ** OR adjusted by centre, age, type of interview and blue-collar status. AIC: Akaike information criterion

Supplementary Table S9 – Number of cases and controls, odds ratio (OR) and 95% confidence interval (CI) by exposure (ever vs never) and cumulative exposure from SYN-JEM, unlagged and at lag 10, 20 and 30 years, men and women – the MISEM study, 2012-2015, Italy. Analyses adjusted also by blue-collar status, as a proxy for socio-economic status.

|  |  |  | **Lag 0** | | **Lag 10** | | **Lag 20** | | **Lag 30** | |
| --- | --- | --- | --- | --- | --- | --- | --- | --- | --- | --- |
| **SYN-JEM exposure index** | **Cases** | **Ctrls** | **OR*** | **CI** | **OR*** | **CI** | **OR*** | **CI** | **OR*** | **CI** |
| **ever/never exposed** |  |  |  |  |  |  |  |  |  |  |
| Unexposed | 278 | 446 | 1.00 | (ref) | 1.00 | (ref) | 1.00 | (ref) | 1.00 | (ref) |
| Exposed | 333 | 262 | 1.84 | (1.37-2.47) | 1.84 | (1.37-2.46) | 1.90 | (1.42-2.55) | 1.77 | (1.33-2.37) |
| *P-Wald test gender interaction* |  |  | *0.22* |  | *0.23* |  | *0.23* |  | *0.49* |  |
| AIC |  |  | 1589.61 |  | 1587.63 |  | 1583.51 |  | 1599.17 |  |
| **below/above median cumulative exposure (in f/mL-y)** | | | | | | | | | | |
| Unexposed | 278 | 446 | 1.00 | (ref) | 1.00 | (ref) | 1.00 | (ref) | 1.00 | (ref) |
| < 0.86 | 120 | 131 | 1.46 | (1.01-2.09) | 1.46 | (1.02-2.10) | 1.57 | (1.09-2.25) | 1.48 | (1.04-2.11) |
| ≥ 0.86 | 213 | 131 | 2.19 | (1.57-3.06) | 2.18 | (1.56-3.05) | 2.21 | (1.58-3.10) | 2.05 | (1.47-2.87) |
| *P-trend* |  |  | *< 0.001* |  | *< 0.001* |  | *< 0.001* |  | *< 0.001* |  |
| *P-Wald test gender interaction* |  |  | *0.40* |  | *0.42* |  | *0.41* |  | *0.70* |  |
| AIC |  |  | 1586.92 |  | 1585.10 |  | 1582.20 |  | 1598.23 |  |
| **tertiles of cumulative exposure (in f/mL-y)** | | | | | | | | | | |
| Unexposed | 278 | 446 | 1.00 | (ref) | 1.00 | (ref) | 1.00 | (ref) | 1.00 | (ref) |
| <0.34 | 64 | 87 | 1.19 | (0.77-1.83) | 1.18 | (0.77-1.82) | 1.28 | (0.84-1.97) | 1.07 | (0.69-1.65) |
| 0.34-1.62 | 118 | 87 | 2.08 | (1.42-3.06) | 2.10 | (1.43-3.08) | 2.15 | (1.46-3.16) | 2.04 | (1.39-2.99) |
| >1.62 | 151 | 88 | 2.21 | (1.52-3.21) | 2.19 | (1.51-3.17) | 2.25 | (1.54-3.27) | 2.19 | (1.50-3.18) |
| *P-trend* |  |  | *< 0.001* |  | *< 0.001* |  | *< 0.001* |  | *< 0.001* |  |
| *P-Wald test gender interaction* |  |  | *0.34* |  | *0.32* |  | *0.27* |  | *0.53* |  |
| AIC |  |  | 1585.89 |  | 1583.92 |  | 1581.18 |  | 1593.40 |  |
| **cumulative exposure, continuos** | | | | | | | | | | |
| Unexposed | 278 | 446 | 1.00 | (ref) | 1.00 | (ref) | 1.00 | (ref) | 1.00 | (ref) |
| Unit exposure: 1 f/mL-y | 333 | 262 | 1.22 | (1.10-1.34) | 1.21 | (1.10-1.34) | 1.22 | (1.10-1.34) | 1.24 | (1.11-1.38) |
| AIC |  |  | 1589.62 |  | 1588.15 |  | 1586.06 |  | 1597.53 |  |
| **log-cumulative exposure, continuos** | | | | | | | | | | |
| Unexposed | 278 | 446 | 1.00 | (ref) | 1.00 | (ref) | 1.00 | (ref) | 1.00 | (ref) |
| Unit exposure : 1 log(f/mL-y+1) | 333 | 262 | 1.79 | (1.40-2.30) | 1.78 | (1.39-2.29) | 1.79 | (1.39-2.30) | 1.83 | (1.41-2.38) |
| AIC |  |  | 1584.43 |  | 1582.86 |  | 1580.79 |  | 1592.67 |  |

* OR adjusted by centre, gender, age, type of interview and blue collar status. AIC: Akaike information criterion

Supplementary Table S10 – Number of cases and controls, odds ratio (OR) and 95% confidence intervals (CI) by modality of exposure, according to the Italian National Mesothelioma Register (ReNaM) classification. Reference category: unlikely/unknown exposure, lag 10 years. Analyses restricted to blue-collar cases and controls (see text) – the MISEM study, 2012-2015, Italy

|  | **Men and women** | | | | **Men** | | | | **Women** | | | |
| --- | --- | --- | --- | --- | --- | --- | --- | --- | --- | --- | --- | --- |
| **Exposure** | **Cases** | **Ctrls** | **OR*** | **CI** | **Cases** | **Ctrls** | **OR**** | **CI** | **Cases** | **Ctrls** | **OR**** | **CI** |
| Unlikely/unknown exposure | 83 | 214 | 1.00 | (ref) | 49 | 151 | 1.00 | (ref) | 34 | 63 | 1.00 | (ref) |
| Occupational, definite or probable | 318 | 103 | 11.7 | (7.77-17.5) | 285 | 92 | 12.5 | (7.94-19.8) | 33 | 11 | 9.11 | (3.14-26.4) |
| Occupational, possible | 55 | 77 | 2.15 | (1.32-3.52) | 44 | 64 | 2.05 | (1.17-3.59) | 11 | 13 | 2.71 | (0.87-8.42) |
| Familial | 28 | 35 | 3.53 | (1.86-6.73) | 9 | 23 | 1.49 | (0.59-3.78) | 19 | 12 | 13.1 | (3.94-43.7) |
| Residential | 23 | 46 | 1.50 | (0.76-2.97) | 10 | 26 | 1.26 | (0.50-3.15) | 13 | 20 | 2.47 | (0.72-8.47) |
| Other non-occupational | 7 | 18 | 1.22 | (0.44-3.36) | 5 | 7 | 3.29 | (0.90-12.0) | 2 | 11 | 0.55 | (0.09-3.33) |
| *P-Wald test gender interaction* |  |  | *0.19* |  |  |  |  |  |  |  |  |  |

* OR adjusted by centre, gender, age, and type of interview; ** OR adjusted by centre, age, and type of interview

Supplementary Table S11 – Number of cases and controls, odds ratio (OR) and 95% confidence interval (CI) by exposure (ever vs never) and cumulative exposure from SYN-JEM, unlagged and at lag 10, 20 and 30 years, men and women. Analyses restricted to blue-collar cases and controls (see text) – the MISEM study, 2012-2015, Italy

|  |  |  | **Lag 0** | | **Lag 10** | | **Lag 20** | | **Lag 30** | |
| --- | --- | --- | --- | --- | --- | --- | --- | --- | --- | --- |
| **SYN-JEM exposure index** | **Cases** | **Ctrls** | **OR*** | **CI** | **OR*** | **CI** | **OR*** | **CI** | **OR*** | **CI** |
| **ever/never exposed** |  |  |  |  |  |  |  |  |  |  |
| Unexposed | 188 | 240 | 1.00 | (ref) | 1.00 | (ref) | 1.00 | (ref) | 1.00 | (ref) |
| Exposed | 317 | 247 | 1.70 | (1.24-2.32) | 1.70 | (1.25-2.33) | 1.76 | (1.29-2.40) | 1.62 | (1.19-2.21) |
| *P-Wald test gender interaction* |  |  | *0.83* |  | *0.80* |  | *0.82* |  | *0.73* |  |
| AIC |  |  | 1229.95 |  | 1228.11 |  | 1225.09 |  | 1236.45 |  |
| **below/above median cumulative exposure (in f/mL-y)** | | | | | | | | | | |
| Unexposed | 188 | 240 | 1.00 | (ref) | 1.00 | (ref) | 1.00 | (ref) | 1.00 | (ref) |
| < 0.86 | 108 | 116 | 1.32 | (0.89-1.95) | 1.33 | (0.90-1.97) | 1.42 | (0.96-2.09) | 1.31 | (0.89-1.93) |
| ≥ 0.86 | 209 | 131 | 2.00 | (1.41-2.82) | 1.99 | (1.41-2.82) | 2.02 | (1.42-2.86) | 1.88 | (1.33-2.66) |
| *P-trend* |  |  | *< 0.001* |  | *< 0.001* |  | *< 0.001* |  | *< 0.001* |  |
| *P-Wald test gender interaction* |  |  | *0.93* |  | *0.92* |  | *0.92* |  | *0.93* |  |
| AIC |  |  | 1227.60 |  | 1225.97 |  | 1223.92 |  | 1330.04 |  |
| **tertiles of cumulative exposure (in f/mL-y)** | | | | | | | | | | |
| Unexposed | 188 | 240 | 1.00 | (ref) | 1.00 | (ref) | 1.00 | (ref) | 1.00 | (ref) |
| <0.34 | 53 | 72 | 1.02 | (0.63-1.64) | 1.01 | (0.63-1.64) | 1.10 | (0.69-1.77) | 0.87 | (0.54-1.41) |
| 0.34-1.62 | 115 | 87 | 1.87 | (1.26-2.79) | 1.90 | (1.27-2.82) | 1.95 | (1.31-2.90) | 1.84 | (1.24-2.74) |
| >1.62 | 149 | 88 | 2.01 | (1.37-2.94) | 2.00 | (1.36-2.93) | 2.04 | (1.39-3.00) | 1.99 | (1.35-2.93) |
| *P-trend* |  |  | *< 0.001* |  | *< 0.001* |  | *< 0.001* |  | *< 0.001* |  |
| *P-Wald test gender interaction* |  |  | *0.64* |  | *0.59* |  | *0.55* |  | *0.75* |  |
| AIC |  |  | 1225.95 |  | 1224.05 |  | 1222.38 |  | 1229.23 |  |
| **cumulative exposure, continuos** | | | | | | | | | | |
| Unexposed | 188 | 240 | 1.00 | (ref) | 1.00 | (ref) | 1.00 | (ref) | 1.00 | (ref) |
| Unit exposure: 1 f/mL-y | 317 | 247 | 1.19 | (1.08-1.32) | 1.19 | (1.08-1.32) | 1.19 | (1.08-1.32) | 1.21 | (1.09-1.36) |
| AIC |  |  | 1227.73 |  | 1226.32 |  | 1224.58 |  | 1232.16 |  |
| **log-cumulative exposure, continuos** | | | | | | | | | | |
| Unexposed | 188 | 240 | 1.00 | (ref) | 1.00 | (ref) | 1.00 | (ref) | 1.00 | (ref) |
| Unit exposure : 1 log(f/mL-y+1) | 317 | 247 | 1.69 | (1.31-2.18) | 1.69 | (1.31-2.18) | 1.69 | (1.31-2.19) | 1.74 | (1.33-2.26) |
| AIC |  |  | 1224.05 |  | 1222.53 |  | 1220.83 |  | 1228.68 |  |

* OR adjusted by centre, gender, age and type of interview. AIC: Akaike information criterion
